# Proline provides site-specific flexibility for *in vivo* collagen

**DOI:** 10.1038/s41598-018-31937-x

**Published:** 2018-09-14

**Authors:** Wing Ying Chow, Chris J. Forman, Dominique Bihan, Anna M. Puszkarska, Rakesh Rajan, David G. Reid, David A. Slatter, Lucy J. Colwell, David J. Wales, Richard W. Farndale, Melinda J. Duer

**Affiliations:** 1 Department of Chemistry, University of Cambridge, Lensfield Road, Cambridge, CB2 1EW UK; 2 Department of Biochemistry, University of Cambridge, Downing Site, Cambridge, CB2 1QW UK; 3 Institute of Infection and Immunity, School of Medicine, Cardiff University, Cardiff, CF14 4XN UK; 4 Present Address: Leibniz-Forschungsinstitut für Molekulare Pharmakologie (FMP) im Forschungsverbund Berlin e.V., Campus Berlin-Buch, Robert-Rössle-Str 10, 13125 Berlin, Germany; 5 Present Address: Northwestern University, 633 Clark St, Evanston, IL 60208 USA; 6 Present Address: University of Calgary, 2500 University Dr. NW, Calgary, Alberta T2N 1N4 Canada

## Abstract

Fibrillar collagens have mechanical and biological roles, providing tissues with both tensile strength and cell binding sites which allow molecular interactions with cell-surface receptors such as integrins. A key question is: how do collagens allow tissue flexibility whilst maintaining well-defined ligand binding sites? Here we show that proline residues in collagen glycine-proline-hydroxyproline (Gly-Pro-Hyp) triplets provide local conformational flexibility, which in turn confers well-defined, low energy molecular compression-extension and bending, by employing two-dimensional ^13^C-^13^C correlation NMR spectroscopy on ^13^C-labelled intact *ex vivo* bone and *in vitro* osteoblast extracellular matrix. We also find that the positions of Gly-Pro-Hyp triplets are highly conserved between animal species, and are spatially clustered in the currently-accepted model of molecular ordering in collagen type I fibrils. We propose that the Gly-Pro-Hyp triplets in fibrillar collagens provide fibril “expansion joints” to maintain molecular ordering within the fibril, thereby preserving the structural integrity of ligand binding sites.

## Introduction

The dominant protein components of the extracellular matrix are ordered fibrillar collagens. These collagens must provide well-defined binding sites for many matrix proteins and cell-adhesion receptors, exemplified here by integrins. These same collagen fibrils also constitute the main mechanical component of the extracellular matrix, constantly subjected to local forces from adherent cells, which induce local collagen molecular movements likely to disrupt collagen–ligand bindings. How the collagen molecular and fibrillar structures are able to fulfil at first sight contradictory ligand binding and mechanical roles is an important question for both biology and materials scientists developing biomimetic implant materials.

Fibrillar collagens are triple-helical proteins and, with the exception of their short N- and C-terminal telopeptides, consist entirely of G-X-Y (or Gly-Xaa-Yaa) triplet repeats where X is most commonly the cyclic imino acid, proline, and Y, hydroxyproline (O/Hyp), a post-translational modification of proline^1–4^. The hydroxylation of a Yaa position P, occurring immediately after synthesis of the collagen precursors, causes the resulting O ring to strongly favour the exo conformation (ring Cγ pointing away from the residue C=O group), which in turn pre-organises the peptide chain structure at the O residues towards the polyproline II helix that is required in each strand of the collagen triple helix; thus, GPO triplets in collagens are widely viewed as being essential for triple helix folding and stabilizing the triple helix structure^5,6^.

**Figure 1:**
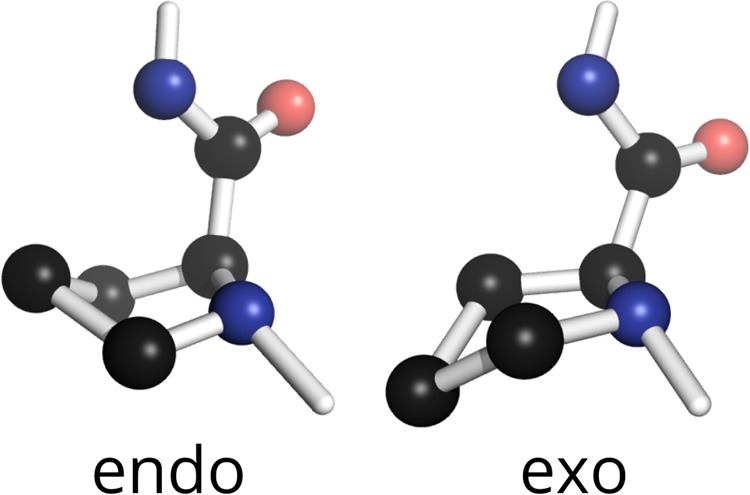
Endo and exo conformations of the proline ring.

The X-position P rings (P_X_) in GPO triplets of short model collagen peptides occupy metastable structures, and endo and exo ring conformations (Fig. 1) are almost equally favoured at biologically-relevant temperatures. Endo and exo conformations have significantly different backbone geometry, and, moreover, flipping between them occurs on a nanosecond timescale^7^. Proline rings in purified collagen preparations are also dynamic on a nanosecond timescale^8–14^; in native hard and soft tissues, both backbone dynamics^15,16^ and proline flipping dynamics^17^ are still retained. Taken together, these data suggest that native collagen GPO triplets are actually flexible, rather than rigid, structural regions. The high GPO triplet abundance in native collagen sequences led us to speculate that GPO P_X_ flexibility could be key to allowing collagen to simultaneously provide essential mechanical properties and structurally well-defined protein binding sites for their biological roles.

## Results and Discussion

### Proline(X) rings are flexible in biologically-derived samples

A significant source of P_X_ flexibility arises from ring flipping between endo and exo conformations. Solid-state NMR spectroscopy provides an accurate method for assessing the distribution of collagen proline ring endo-exo conformations in intact tissues. In previous work, we utilised the ^13^C chemical shift of proline Cγ to determine the distribution of proline P_X_ ring endo-exo conformations in model collagen peptides^7^. There, we deduced that the collagen GPO P_X_^13^C shift is about 24 ppm for the endo conformation, and 25.7 ppm for the exo conformation. Proline rings in crystalline proline^18^ and in model collagen peptides^7^ rapidly flip between endo and exo conformations at room temperature and above, while by measuring dipolar coupling order parameters in native collagen, proline rings have been shown to undergo greater angular fluctuations^17^ compared to glycine and hydroxyproline^13^. Rapid P_X_ ring flipping means that the observed ^13^Cγ isotropic shift is a population-weighted average of the endo and exo conformation ^13^Cγ chemical shifts, allowing the endo:exo population ratios for P_X_ rings to be determined from the population-weighted average shift^7^. In our previous work, we have demonstrated the presence of rapid endo-exo flips for proline rings in model collagen-like peptides containing the GPO/POG motif at room and physiological temperatures, and our simulations indicate that these puckering motions are coupled to the backbone motion of the triple helix. In the present work, we look for evidence of similarity in proline conformation dynamics between model peptides and more biologically-relevant samples to extend our findings to native collagen fibrils in the extracellular matrix.

Resolving the GPO proline ^13^Cγ signals for model collagen peptides where specific ^13^C labelling is readily achieved is straightforward. However, resolving this signal in intact tissue samples is not as easy; at natural ^13^C abundance, the ^13^C NMR spectrum of an intact collagenous tissue contains signals from all amino acid residues, including collagen GPY triplets, where Y is any residue not hydroxyproline (O), as well as the GPO proline signals of interest. Here we use ^13^C, ^15^N-enriched mouse bone and *in vitro* sheep osteoblast matrix, both generated as described previously^19,20^, as models for fibrillar collagen type I in intact tissues. 1D NMR spectra of all samples reported in this manuscript can be found in Supplementary Fig. S2.

**Figure 2:**
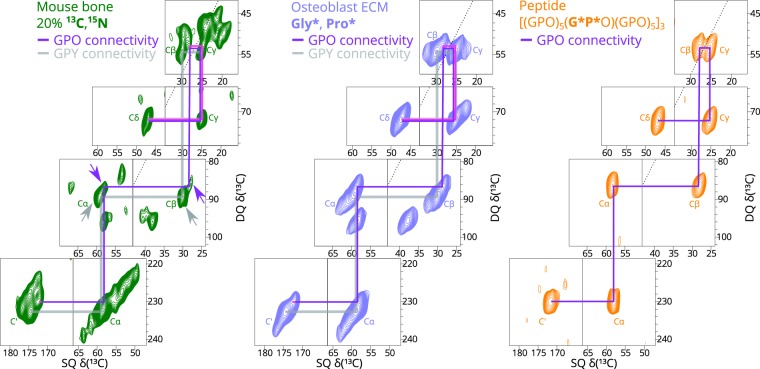
2D ^13^C-^13^C DQ-SQ correlation spectra of mouse calvarial bone (green), *in vitro* osteoblast extracellular matrix (blue) and model collagen peptide (orange). All spectra were obtained at 10 kHz MAS and 297 K, and only the proline carbon regions are shown in this figure (full spectra can be found in Supplementary Fig. S3). The peptide and osteoblast ECM samples are isotopically enriched specifically in glycine and proline residues. In the bone sample, ~20% of essential amino acids and glycine are U-^13^C, ^15^N labelled. The GPO proline signals are labelled in the figure and the ^13^C-^13^C correlations between them are traced (purple line) for the model peptide spectrum, starting with the amide carbon at around 172 ppm. The same trace is overlaid on the bone and osteoblast matrix spectra to show how we arrived at the assignment of the collagen GPO proline signals in those spectra, with a slight difference in that the Cγ signal in the spectra obtained from biologically-derived samples is at 24.8 ppm rather than 25.3 ppm in the peptide spectrum (pink line). The GPY (where Y ≠ O) connectivity is shown to be separate from that of GPO (grey line). The arrows on the bone spectrum indicate the two sets of signals corresponding to Cβ-Cγ, where the difference between the GPO (purple) and GPY (grey) signals can be most clearly observed. The black dotted line in each spectrum indicates the DQ-SQ diagonal.

Isotopic enrichment is necessary to use two-dimensional ^13^C NMR spectroscopy for resolving the proline ^13^Cγ chemical shift distributions in these intact tissues through ^13^C-^13^C double-quantum–single quantum (DQ-SQ) correlation spectra. In this type of spectrum, pairs of strongly dipolar-coupled (i.e. spatially close) ^13^C nuclei give signals in the double-quantum spectrum at the sum of the chemical shifts for the two ^13^C nuclei. These DQ signals are correlated in the other spectral dimension with the normal (single) quantum ^13^C NMR spectrum for the respective pair of ^13^C nuclei. Horizontal slices through the DQ-SQ correlation spectra thus yield one-dimensional ^13^C spectra of pairs of spatially close carbon nuclei. 2D ^13^C DQ-SQ spectra from three ^13^C, ^15^N-labelled collagen/collagen-like samples are shown in Fig. 2 for comparison: mouse bone, cultured osteoblast matrix and a model triple-helical collagen peptide, ((GPO)_5_(G*P*O)(GPO)_5_)_3_, where * indicates a U-^13^C, ^15^N-labelled residue (in each and every chain of the triple helix). As Fig. 2 shows, the connectivity of bonded carbons in the proline ring can be traced out in the DQ-SQ spectrum. By comparison with the signal connectivities in the model peptide spectrum, we can make straightforward assignment of collagen proline P_X_ signals in the ^13^C, ^15^N-labelled mouse bone and *in vitro* matrix spectra.

We then need to separate ^13^C signals from P_X_ in general GPY triplets, where Y ≠ O (233 per collagen triple helix) from those in GPO triplets (103 per triple helix) in the mouse bone collagen DQ-SQ spectrum. We utilise the fact the ^13^C signals from P_X_ in GPO triplets are subject to the proline effect^21,22^, which manifests as a 1–2 ppm reduction in ^13^C chemical shift for Cα, Cβ and C′ for residues that precede an imino acid, as in the case of P_X_ in GPO triplets, where P_X_ precedes Hyp/O, but not for P_X_ in GPY triplets. The GPO P_X_ Cα-Cβ correlation in the bone DQ-SQ spectrum can be distinguished from the corresponding GPY P_X_ correlation using this criterion, as indicated in Fig. 2.

**Figure 3:**
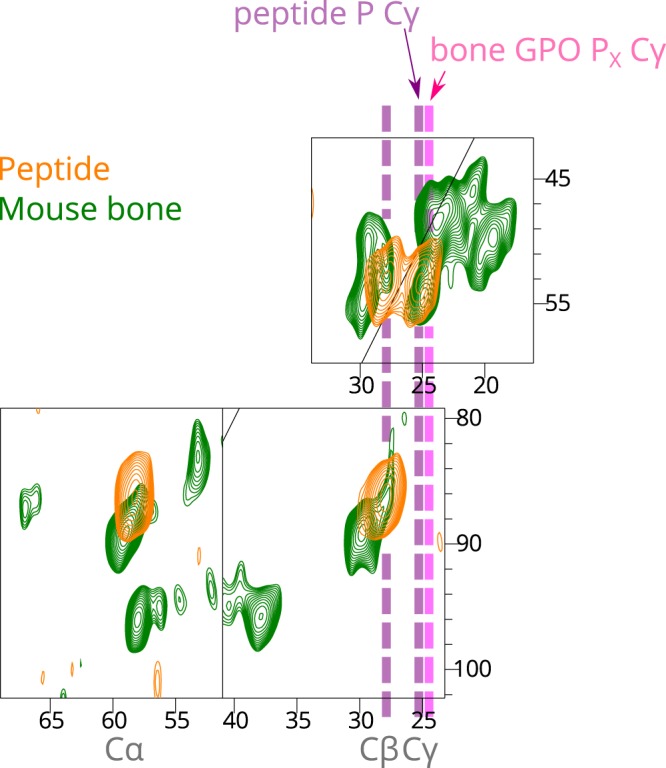
Overlay of the 2D ^13^C-^13^C DQ-SQ correlation spectra of mouse calvarial bone (green) and model collagen peptide (orange), emphasising the identification of the GPO P_X_ population in the mouse bone spectrum.

From the GPO P_X_ Cα-Cβ correlation, the GPO P_X_ Cβ-Cγ correlations are found, as shown in Fig. 2 and Fig. 3, from which the mean ^13^Cγ chemical shift for the bone GPO P_X_ rings is determined as 24.8 ppm, with partial overlap with the main population of GPY centred at 25.3 ppm. We note that the strong similarity of the proline signal chemical shift distribution between the mouse bone and *in vitro* matrix samples for all proline signals is a clear indication that the collagen structures in the two samples are similar, unlike the peptide proline distribution, which clearly only overlaps with a subset of that found in bone.

As discussed above, this ^13^Cγ chemical shift represents a population-weighted average between the ^13^Cγ chemical shifts expected for the endo (23.8 ppm) and exo (25.7 ppm) proline ring conformations^7^. The mean ^13^Cγ chemical shift of 24.8 ppm for bone collagen GPO P_X_, while only a small change compared to the ^13^Cγ of 25.3 ppm for the (GPO)_11_ model collagen peptide^7^, indicates the mean population distribution for collagen GPO P_X_ in the intact tissues is slightly more skewed towards endo compared to the collagen model peptide. Nonetheless, this observed ^13^Cγ chemical shift (24.8 ppm) is still distinct from a “pure” endo chemical shift (23.8 ppm), indicating that the proline rings in GPO triplets in bone do not strongly favour the thermodynamically more stable endo conformation^23–25^, and instead show a distribution between endo and exo conformations, with fast exchange over the NMR time scale, i.e. they are flexible.

**Figure 4:**
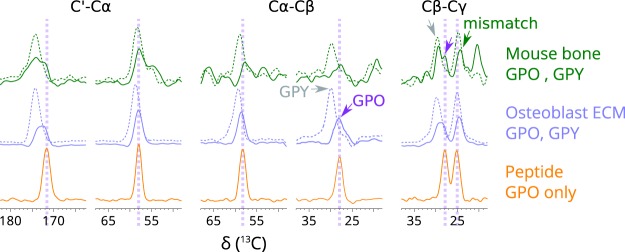
Slices taken from the DQ-SQ correlation spectra of mouse calvarial bone (green), *in vitro* osteoblast matrix (blue) and model collagen peptide, showing the ratio of GPO to GPY (where Y ≠ O) signals. GPO slices are shown with solid lines (some with purple arrows), GPY slices are shown with dashed lines (some indicated with grey arrows). The Cγ assignment in Fig. 3 is shown with a green arrow on the bone GPO Cβ-Cγ slice. Vertical scaling was normalised on the larger signal within a GPO/GPY pair, maintaining the intensity difference between GPO and GPY to scale, but the vertical scale between pairs of GPO/GPY and between different samples are not shown to a unified scale. All spectra are presented on the same horizontal scale, enabling comparison of the chemical shift and also the linewidth at half height.

To confirm our assignment, we note that the intensity of the signals arising from GPO triplets compared to the GPY (where Y ≠ O) should match the expected ratio in the sequence for mouse collagen type I. Figure 4 shows 1D slices taken from the SQ-DQ experiment in Fig. 2, at the C′-Cα, Cα-Cβ, and Cβ-Cγ DQ (sum) frequencies. The GPY:GPO triplet ratio in the sequence is 233:103, which means we expect to observe a GPY signal that is approximately 2.3 times more intense than that of GPO (assuming similar linewidths, if not then the integral of the signals should match this ratio). From Fig. 4, we can see that this is indeed true. The effect is clearer in the case of the osteoblast matrix cultured *in vitro* than the bone spectrum, as the bone spectrum is labelled in other amino acids apart from proline and glycine, giving rise to overlapping signals that can skew the observed ratio.

We expected that GPO P_X_ would occupy a much larger range of conformations for fibrils in intact tissues compared to model collagen-like triple helical peptides. However, this is not the case, as evidenced by the NMR lineshapes for different samples shown in Fig. 4. The relative line widths are most easily assessed for the model peptide and *in vitro* osteoblast matrix, because the Pro ^13^C signals are not obscured by signals from other labelled amino acids as they are for the bone sample.

There is some overlap between GPO and GPY proline ^13^C signals for the osteoblast ECM sample, particularly for C′, which needs to be taken into account when comparing the Pro ^13^C lineshapes (GPY C′ sites give a shoulder on the high frequency side of the GPO C′ signal). Nevertheless, it is clear that the GPO proline Cα and Cβ lineshapes are highly similar between model peptide and *in vitro* matrix collagen. The Cα lineshapes are directly comparable. In the Cβ spectrum, there is some additional small signal to low frequency for the *in vitro* matrix sample, but the majority signal has a similar linewidth to that for the model peptide.

The lineshapes allow us to directly compare the range of conformations for GPO P_X_ between collagen in an intact tissue and the model collagen peptide. The single-quantum NMR lineshapes are in effect the sum of individual lineshapes from each GPO P_X_ residue in the sample, each having an isotropic shift determined by the local molecular geometry for each individual P_X_. These individual lineshapes are not resolved, but the width of their sum signal, i.e. the observed signal in the SQ dimension of the DQ-SQ spectra, retains information on the range of GPO P_X_ isotropic chemical shifts and thus on the distribution of time-averaged P_X_ ring conformations.

Apaft from the distribution of proline ring geometries, the NMR linewidths can be affected by by other factors: T_2_ (transverse) relaxation, homonuclear ^13^C-^13^C dipolar coupling and molecular motion all contribute to the homogeneous linewidth. Hydration and sample temperature also play a role. While we have carried out our experiments with a fairly low level of hydration in order to capture a maximum range of conformations (lyophilization generally traps conformations and increases heterogeneous broadening), and maintained them at similar experimental temperatures, we cannot resolve the different contributions to the ^13^C lineshapes. However, correspondence of the overall ^13^C lineshapes implies some similarity in the distribution of proline residue conformations, molecular motion and T_2_ relaxation time (which itself depends on molecular motion).

We further confirmed similarity in the distribution of GPO geometries between the *in vitro* matrix collagen and the collagen model peptide by following the build-up of G-P ^13^C correlation signal intensity in a series of 2D ^13^C-^13^C proton-driven spin diffusion (PDSD) correlation NMR experiments in which the mixing time for ^13^C to ^13^C magnetization transfer is varied (see Supplementary Fig. S4).

In summary, we see that P_X_ in GPO exhibits a mix of endo and exo conformations, in a distribution that is similar to what we see in the model peptide. In our previous work^7^, we have shown that collagen-like model peptides exhibit a dynamic equilibrium of endo and exo conformations, with the equilibrium positioned at a ratio of near 50:50 at biologically-relevant temperatures. With the experimental data summarised thus far, we propose that the same distribution of endo and exo conformations (with very little bias towards endo) is observed in biological samples.

### The Pro(X) rings in GPO triplets have greater local conformational flexibility than in GPY sequences

From the NMR data presented above, we determined that P_X_ in GPO in native collagen samples has a distribution over endo and exo conformations. However, it is also clear that P_X_ in GPY (where Y ≠ O) exhibits a ^13^Cγ chemical shift that is also greater than that in a pure endo case. In previous work^7^, we used the potential energy landscape approach^26^ to demonstrate the inherent flexibility or frustration of helix parameters, which is coupled to P_X_ endo-exo flips in GPO sequences. We use the same approach here to analyse whether the flexibility of P_X_ rings in GPO triplets differs in any way from GPY triplets, where Y is a conformationally less constrained amino acid. Specifically, we predict the structural behaviour of P_X_ rings in a GPA triplet compared with those in a GPO triplet. A GPA triplet was selected since it is the most common collagen type I GPY triplet in which Y is an amino rather than an imino acid.

All the calculations involve geometry optimisation to characterise local minima and the transition states and pathways that connect them on the potential energy landscape. By applying appropriate structural perturbations to a ground state conformation, this procedure allows for sets of energy minima to be computed that span particular regions of conformational space. For the present purposes we are interested in the subspace of minimum energy conformations that can be adopted by perturbing the backbone dihedral angles of a single GPY triplet while it is embedded within in a larger collagen triple helix. Flexibility at a residue comes from the presence of a range of energetically-accessible conformations for that residue. The relative number of distinct minima found in each subspace and the corresponding range of dihedral angles are taken as a measure of the likely flexibility. If many alternative conformations exist, separated by thermally accessible barriers on the experimental time scale, then we predict greater flexibility.

The structural perturbations to the ground state structure were carefully designed to uniformly sample the space of backbone dihedral angles and proline endo and exo conformations for the sixth triplet in the trailing chain in both (POG)_12_ and (PAG)_12_ superstructures. A single perturbation consisted of choosing a pair of atoms separated by a linear set of covalent bonds, and rotating as a rigid body all the intervening atoms by an angle randomly selected within a maximum amplitude. When such rotations occur between two backbone atoms the local ring geometry and endo/exo structures are conserved, but the backbone dihedrals, in which the atom pair are involved, change. Rotations applied to the atoms between the Cβ and Cδ atoms in the proline rings can force a subsequent relaxation into either endo or exo conformations, depending on the angle of rotation, which also modifies the backbone dihedrals of the proline and neighbouring residues. Further details of the particular groups considered can be found in the Methods.

**Figure 5:**
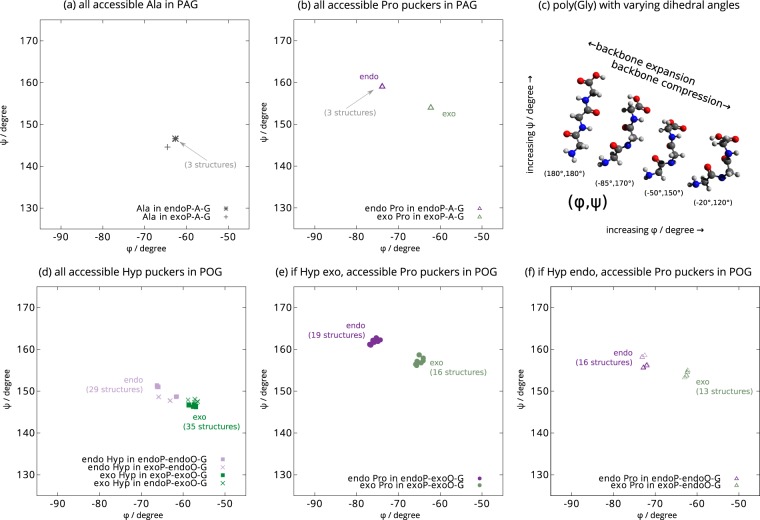
The energy landscape simulation results presented as Ramachandran plots. For each different, accessible conformational structure, the dihedral angles of two consecutive residues, proline and the residue subsequent to proline, are plotted. These backbone conformations arise from perturbing a single Pro residue in (PAG)_12_ and (POG)_12_ whilst maintaining the overall ring conformation as either endo or exo. For (PAG)_12_, only four accessible structures were found, three of which were very similar in backbone dihedral angles in the current scaling, and therefore overlap nearly perfectly. (**a**) shows the backbone dihedral angles for the alanine residue of these four structures and (**b**) shows the backbone dihedral angles for the proline residues, showing a clear separation in backbone dihedral angles for endo and exo ring conformations. For (POG)_12_, 64 accessible structures were found. (**d**) shows the backbone dihedral angles for the hydroxyproline for all 64 structures, split into two populations according to the conformation of the hydroxyproline ring. The preceding proline ring conformations were plotted for the case where hydroxyproline is exo (**e**) and endo (**f**). Panel (c) illustrates the dihedral angle change in terms of overall backbone conformation using structures generated for a GGG tripeptide (glycine was used for clarity) of constant dihedral angle using Avogadro 1.1.1^54^, ranging from a fully extended backbone conformation (180°, 180°) to a much more compressed coiled conformation (−20°, 120°). It is clear that as ϕ and ψ increase, the peptide backbone is increasingly extended, while as the dihedral angles tend towards lower values, the backbone is increasingly compressed. Although the dihedral angle changes presented in the simulation represent a level of change that is smaller than that shown in the middle two peptides in (**c**) (a length difference of under 5%), the absolute extent of expansion/compression will scale with the length of the peptide. As previously reported^7^, the idealized 7/2 (tighter) and 10/3 (looser) triple helices both have dihedral angles that more closely match endo P_X_ in (**b**,**e** and **f**).

By repeating our structural perturbations for each triplet, an ensemble of feasible conformations, corresponding to local potential energy minima, were generated. This analysis yielded only four minima in the case of (PAG)_12_, and 64 minima in the case of (POG)_12_. Rather than presenting the whole database of structures, which would be unwieldy, we illustrate the backbone dihedral angles of two neighbouring P_X_ and Y residues in the centre of the sequence for each structure in a Ramachandran plot (Fig. 5). For ease of viewing, each residue (proline and alanine, or proline and hydroxyproline) is presented on different panels of the figure. In the case of P_X_ in (POG)_12_, we have further divided the plot into two, based on whether the hydroxyproline in the structure has an endo or an exo pucker. Thus, each point in each Ramachandran plot represents a minimum in the potential energy landscape, i.e. an alternative accessible conformation.

For P_X_ rings in the GPA triplet, all the perturbed minima in the case of P_X_ endo pucker have nearly identical dihedral angles, resulting effectively in just two possible backbone conformations, one associated with endo pucker at P_X_, and the other with exo pucker. There are therefore, two well-defined, accessible backbone conformations at GPA triplets. To deform the GPA triplet, or in other words, to access GPA P_X_ backbone structures with significantly different ϕ/ψ angles to these endo or exo minima, would require large energy perturbations, corresponding to thermally inaccessible barriers at biologically relevant temperatures.

In striking contrast, the P_X_ ring in a GPO triplet can move between local energy minima that exhibit a range of ϕ/ψ angles for both endo and exo P_X_ conformations. The diversity of conformations accessed via local structural perturbations for GPO P_X_ rings depends on whether the neighbouring Hyp is in the endo or exo conformation. The combination of endo and exo conformations for proline and hydroxyproline in GPO gives rise to four clusters, as illustrated in Fig. 5(d,e), where P_X_ (and Hyp) endo-exo ring conformations are accompanied by systematic shifts in backbone conformation corresponding to extension-compression of the helical chain.

Combined with the NMR results presented in the first section, we conclude that the GPO proline rings in native collagen proteins exhibit flexibility in the form of endo-exo pucker, just as previously observed in model peptides. Our computational results further demonstrate that these endo-exo pucker motions are not limited locally at the proline ring, but are coupled to the backbone, and can lead to overall extension and compression of the triple helix at GPO triplets. In the context of heterotrimeric collagens and fibrils formed from collagen triple helices, it is likely that these extension and compression motions will lead to bending of the triple helix and the fibril.

### GPO Pro(X) flexibility may be necessary for the biomechanical function of collagen

We next asked what is the role of such flexibility at GPO triplets, and specifically whether it could impact on fibril as well as molecular flexibility. To determine the location of the evolutionarily conserved GPO triplets in the fibril, we carried out sequence alignment across diverse species for collagen type I and generated a consensus sequence for each chain. Using the database of collagen type I sequences, we also calculated the conservation of each GPO triplet. Full results of the calculations for collagen type I are presented in Supplementary Table S1 and Fig. S5.

From our sequence analysis, we find that there are 33 highly conserved GPO sites across collagen α1(I) chains out of 43 in the consensus sequence, and 20 highly conserved GPO triplets across collagen α2(I) chains out of 33 in the consensus sequence. If we consider only mammalian sequences, these values increase to 41 out of 43 GPO triplets in the α1(I) chain, and 24 out of 33 in the α2(I) chain being defined as highly conserved at the same conservation threshold (75%).

**Figure 6:**
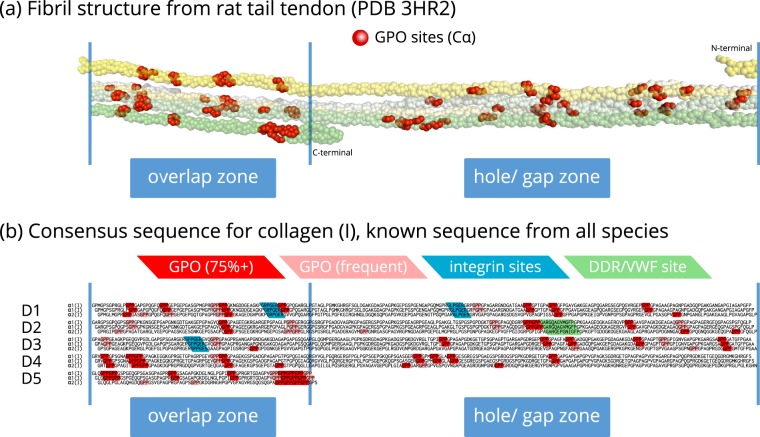
The distribution of GPO sites within a type I collagen fibril, from diffraction data (**a**) and in the consensus sequence (**b**). In (**a**), the collagen fibrillar structure (from PDB 3HR2^27^) is modelled from X-ray diffraction data on rat tail tendon^28^ with the Cα atoms in GPO triplets represented as red spheres. In (**b**) the consensus sequence based on the most frequent amino acid at each position is shown. The three chains of collagen are staggered by one residue with respect to each other, based on a previous study on the VWF A3 domain binding^55^. The D period arrangement shows a good match to the experimentally-derived fibril structure model above. Highly conserved (75%+) GPO triplets are highlighted in red; others in pink. High affinity integrin binding sites are in blue and the DDR/ VWF binding site in green.

The distribution of GPO sites in the experimentally-determined model^27^ of collagen type I fibrils and also the consensus sequence is shown in Fig. 6. For ease of comparison, the consensus sequence for the collagen type I α1 and α2 chains is arranged by D-period. The consensus sequence for type I collagen is provided as a larger PDF image in the Supplementary data.

From Fig. 6, it is clear that there is a spatial correlation of GPO sequences; far from being randomly scattered over the fibril structure, these sequences occur primarily as banded clusters across the fibril structure that persist across different D-periods, exemplified in the XRD-derived collagen type I structural model^28^ in Fig. 6a and the sequence representation in Fig. 6b.

The distribution of GPO triplets varies within the fibril structure. Collagen fibrils have two zones: the overlap zone, where all five D periods overlap, and the hole zone, where the short D5 period leads to a gap or hole in the fibrillar structure. There are clearly more GPO triplets present in the overlap zone, and fewer in the hole/gap zone. The banding of GPO triplets is also more obvious in the overlap zone.

Bearing in mind the conformational flexibility of the GPO triplet structure, these clusters of GPO triplets in the fibril represent regions where the fibril structure itself can rapidly extend, compress or bend, and so serve as flexible joints for both the molecular and fibrillar structure, without significantly increasing the energy of the system. Hence, we propose that these GPO triplets act as “expansion joints”, especially when viewed in the context of their distribution over the fibril. While Y-position hydroxyproline is known to increase the thermal stability of a collagen triple helix^29^, and to promote PPII formation in the backbone^5,30^, the secondary effect of placing the preceding proline into a conformationally frustrated state (i.e. with many pucker conformations of similar energy)^7^ provides local flexibility at these conserved locations of the triple helix and therefore also of the fibril. Such local flexibility can be important for the overall integrity of the fibril, thus explaining the observation that thermal stability is associated with total imino acid content (proline and hydroxyproline) and not hydroxyproline alone^31^.

We consider three important implications arising from our proposal of GPO sites as expansion joints in collagen fibrils: firstly, the role of of GPO triplets in collagen binding by considering the distribution of GPO relative to known binding sites on collagen proteins; secondly, the possibility that asymmetrical distribution GPO triplets may aid larger angle bending and functional displacement of the C terminus in the D5 period; and thirdly, the consequences of known mutations of conserved GPO sites.

### Role of GPO triplets in binding of collagen fibrils to other ECM components

Different types of binding are likely to exert different mechanical forces on the collagen fibril. The integrin α2β1 domain–peptide co-crystal (PDB 1DZI)^32^ describes a bend in the model collagen peptide upon ligation, suggesting that molecular distortion may be necessary for optimal interaction of native collagen with the integrin. Such molecular distortion upon binding to the integrin would affect the integrity of the collagen fibril, and if replicated many times by the multiple collagen-integrin interactions presented by a cell, significant disorder of the collagen fibril would result.

We note that the high affinity integrin binding sites, shown in Fig. 6, all occur in close proximity and on the N-terminal side of conserved GPO triplets. On the other hand, the main DDR and VWF binding site, also occurring close to multiple strongly conserved GPO triplets, is found on their C-terminal side. We hypothesise that the clusters of GPO triplets serve to protect the structural integrity of the adjacent sites when the fibrillar structure is subjected to external forces. The multiple highly conserved GPO triplets close to the DDR/VWF binding site in the fibril (as seen in the D-period arrangement of the consensus sequence in Fig. 6b) may provide additional controlled flexibility for this binding site.

We speculate that local structural distortion is necessary to maintain the organization of collagen molecules within the fibrillar structure when ligands bind, and that clusters of GPO triplets across the fibril in close proximity to the collagen-ligand binding site provide controlled, reversible local distortion of the structure.

### Asymmetrical distribution of GPO triplets may facilitate functional bending of the triple helix

The distribution of GPO triplets between the three chains of the collagen triple helix can affect its accessible overall motion range. Where a GPO sequence occurs at the same locus of all three chains, proline ring endo-exo flips will allow extension-compression in all three chains, permitting concerted movement at this position. The stagger between the three chains dictates that extension-compression is never restricted to the axis of the triple helix, but will always include a bending component.

For collagen type I, a heterotrimer containing two α1 chains and one α2 chain, there are many cases where GPO triplets do not occur at the same locus in both the α2 and α1 chains. Chain extension-compression can then only occur asymmetrically, bending the triple helix in a specific direction, through an angular range defined by the change in chain backbone structure upon proline endo-exo ring flip, typically a change of around 15° in proline dihedral angle.

Interestingly, the majority of the GPO triplets in the fibril hole zone only occur in either the α1 or α2 chains. The X-ray fibre diffraction-derived model of the collagen fibril structure^27^ shows the collagen triple helices twisting around one another through the hole zone. If this molecular twisting were to occur via homogeneously flexible regions in the collagen triple helices, molecular ordering would be compromised, as there would be no control over which direction (or by how much) a collagen triple helix can bend. However, an appropriate distribution of GPO triplets within the hole zone could readily allow well-defined bending in a specific direction, which would allow the collagen triple helices to twist around one another without the possibility of molecular disordering. Such considerations, exemplified here by collagen I, will not apply to the homotrimeric fibrillar collagens, II, III, XXIV and XXVII, but will be relevant to the heterotrimeric collagens V and XI.

The (GPO)_5_ and (GPO)_4_ sequences at the C termini of the α1 chains and α2 chains, respectively, are well-conserved in the fibrillar collagens. The triple helix extension-compression ability that these sequences confer would allow the C-terminus some flexibility in its spatial location, thus our hypothesis that the GPO sequences play the role of “expansion joints”. All other GPO triplets in D5 that precede the (GPO)_5_ sequence occur only in the α1 chains, so that D5 has controlled freedom to bend at these three points. It has previously been hypothesized that the C-terminal telopeptide protects underlying ligand binding sites in the collagen fibril molecular structural arrangement^33^; the (GPO)_5_ sequences and D5 period that precede it in primary sequence may assist in such a function, by controlling the possible displacements of the C-terminal, but at the same time allowing it to be displaced, thus allowing ligand binding.

### GPO clusters can tolerate pathological mutations in collagen type I

While the GPO positions are evolutionarily conserved, we assessed the known mutations at these sites that lead to human disease. Using the NCBI ClinVar database^34^, we assessed missense mutations in collagen, and the overlap of these mutations with locations of conserved GPO triplets, to understand the functional and likely pathological consequences. We are aware that this type of analysis is prone to survivor bias. With this caveat, we will offer a few interpretations of our results.

As far as the collagen type I triple helical region is concerned, there are 95 unique single base mutations for the α1(I) chain, and 110 for the α2(I) chain. For α1(I), we found 33 highly conserved GPO sites in the consensus sequence, of which 10 correspond to the reported mutation sites (human variants). Conserved GPOs account for 9.8% of the total helix sequence, and 10.5% of their locations overlap with possible mutation sites. For α2(I) we found that there are 9 GPO locations out of 20 highly conserved that overlap with reported mutation sites. 5.9% of the chain consists of conserved GPO triplets, and 8.2% of the 110 mutations overlap with conserved GPOs.

By inspecting the sites where mutations overlap with bands or clusters of conserved GPOs, we note that 16 mutations (in both chains α1(I) and α2(I)) occur within a GPO band/cluster, while six mutations occur in relatively isolated GPOs. However, given the fact that many more GPO triplets occur in clusters within the triple helix or across the fibril, and relatively few are isolated, it is difficult to draw firm conclusions from this observation.

The diseases associated with the reported mutations appear to vary by chain. Most mutations in α1(I) are associated with osteogenesis imperfecta, and in α2(I) with Ehlers Danlos syndrome.

These results show that although mutations occurring in GPO triplets in collagen type I can be tolerated, in the sense that it is possible to survive past embryonic developmental stages, they lead to pathology in most cases. A more detailed analysis including more collagen types will be required to separate the effects of GPO (or lack thereof) on biomechanical properties of the fibril and its effects on biosynthesis and embryonic development.

## Methods

### Peptide synthesis and purification

((GPO)_5_(G*P*O)(GPO)_5_)_3_ was synthesized as previously reported^7^. Briefly, the peptide was synthesized (0.1 mmol scale) as C-terminal amides on a TentaGel R RAM resin (loading of 0.19 mmol/g, Rapp Polymere) following the standard Fmoc-based solid-phase peptide synthesis strategy on a microwave-assisted automated peptide synthesizer (Liberty™, CEM), and purified by reverse phase HPLC. Pure peptides were characterised by matrix-assisted laser desorption and ionization-time of flight (MALDI-TOF) mass spectrometry (Supplementary Fig. S1).

### Labelling of mouse tissue

Our feeding and euthanasia methods were unregulated procedures under the UK Animals (Scientific Procedures) Act 1986 and therefore were not subjected to formal ethics review. Our methods complied with the review processes of the University Biomedical Service of the University of Cambridge, which is overseen by the Animal Welfare Ethical Review Body of the University of Cambridge. All experiments were performed in accordance with the UK Animals (Scientific Procedures) Act 1986.

The procedure is based on our previous work^19^. Briefly, *in vivo* labelling was achieved with 2000 g of a gel diet (modified Classic A03 Geldiet, SAFE, Augy, France) to minimize in-cage spillage, comprising 50 g ^13^C, ^15^N-labelled Celtone powder (Cambridge Isotope Laboratories, Andover, MA, U. S. A.), 67 g fish hydrolysate, 410 g protein free diet (mainly corn starch), 72.9% water, 2.1% preservatives and texture additives, which was packaged in 100 g packs and irradiated at 10 kGy. Three young adult female C57Bl/6 mice, housed together, were fed ad libitum for ca. 3 weeks until the labelled diet was consumed, humanely euthanized using a Schedule 1 method and tissues harvested. Bone tissues from all animals were examined by solid-state NMR to ensure we were not assigning small biological variations between animals, and did not show major variation.

### Isolation of fetal sheep osteoblasts

Fetal sheep osteoblasts were isolated from a fetus removed from an 18 weeks pregnant sheep sacrificed for an unrelated study. Femurs were removed from the fetus. After washing several times with 1% trigene (Medichem International), the femur was stripped of muscle and non-osseous tissue to expose the bone which was sectioned into small longitudinal pieces and washed with 70% ethanol followed by repeated washings with Minimum Essential Medium (MEM; Invitrogen) to remove all traces of ethanol. Bone strips were then transferred to Dulbecco’s Modified Eagle Medium (DMEM; Invitrogen) containing bacterial collagenase A (0.5 mg/mL) and dispase II (3 mg/mL) both from Roche Diagnostics. A total of 100 mL of enzyme-media mixture was used for bone sections taken from 3 limbs. Bone strips were incubated at 37 °C in a shaking water bath for 3 hours to release osteoblasts into the medium. After incubation the cell suspension was transferred to a fresh tube and the bone sections were rinsed in DMEM with 20% fetal calf serum (FCS; Invitrogen) to stop the enzymatic digestion. Rinse medium and cell suspension were pooled and passed through a 40 μm mesh filter (Appleton Woods). The cell suspension was then centrifuged at 1000 g for 5 min at room temperature to pellet the cells. The pellet was resuspended in DMEM complete medium and transferred to two T-175 cm^3^ culture flasks (Nunc) and placed in a 37 °C CO_2_ incubator. When the cultures were almost confluent, cells were detached with 10 ml of 0.25% trypsin containing 1 mM EDTA (SigmaAldrich) and incubated for 5 min at room temperature. The flasks were tapped at the end of incubation period to completely dislodge the cells from the flask. Trypsin was neutralized by adding 15 mL of DMEM complete media to the culture flask. The cell suspension was centrifuged in a 50 mL tube (Greiner) at 1200 rpm for 5 min and resuspended in 10 mL of DMEM. The cells were transferred into T-175 cm^3^ culture flasks and were expanded to passage 3 for subsequent experiments.

Basal Medium Eagle (BME) complete medium was prepared by adding 10% FCS, 30 μg/mL L-ascorbic acid 2-phosphate (Sigma), 10 mL/L L-glutamine-penicillin- streptomycin (200 mM L-glutamine, 10,000 units/ml penicillin, and 10 mg/ml streptomycin in 0.9% sodium chloride; Sigma). DMEM complete medium was prepared by adding 10% FCS, 30 μg/mL L-ascorbic acid 2-phosphate, and 10 mL/L L-glutamine-penicillin-streptomycin. All supplements were filter sterilized (0.22 μm filter, Appleton Woods) before addition.

### Culturing osteoblasts with labelled compounds

The procedure is based on our previous work^19^. Briefly, osteoblasts were cultured to confluence in T-175 flasks containing 25 mL BME complete medium. Labelled (U-^13^C_5_, ^15^N) proline (Cambridge Isotope Laboratories) and (U-^13^C_2_, ^15^N) glycine (Cambridge Isotope Laboratories) were added to a final concentration of 46 mg/L and 30 mg/L respectively after filter sterilization (0.22 μm filter). The cultures were incubated at 37 °C in a humidified atmosphere of 95% air and 5% CO_2_. The culture medium with isotope labelled supplements was renewed every two days until the cells and matrix began to detach from the culture flask, by which time enough ECM had formed for solid-state NMR. Refinement of the cell culture method to produce ECM that was a similar as possible to the native mouse bone tissue as judged by their respective 2D solid-state NMR spectra included adjusting the cell culture medium, the frequency with which the medium was changed, the concentration of ascorbic acid and the manner in which the cultures were handled (so as to produce minimal shear forces on the cells during changes of medium produced the optimal ECM). Samples from more than 20 batches using the final optimized protocol were prepared using isotope-enriched amino acids and all characterized by solid-state NMR to ensure reproducibility of results.

### Harvesting ECM from cell culture

The matrix was harvested after nine days of culture, when the cells produce a dense matrix which started to peel off the surface of the tissue culture flask. The medium was removed and the cells were washed with 20 mL 1 x phosphate buffered saline. The flask was placed in a freezer at −80 °C for 24 hours and the cells were lysed by thawing the flasks at room temperature for 30 minutes. The debris produced by cell lysis was removed by repeated washes with PBS. The decellularized ECM was dislodged by gently swirling the flask in the presence of 20 mL PBS. The matrix collected in PBS was transferred to a fresh 50 mL tube and centrifuged at 1200 rpm for 5 min at room temperature. The supernatant was poured off and the ECM dehydrated in an oven at 37 °C overnight. The samples were stored at −20 °C until NMR analysis. ECM of mouse tissue was used directly in solid-state NMR experiments without extraction, purification, or excessive processing.

### Rotor packing for solid-state NMR experiments

All samples were placed into Kel-F inserts for Bruker 4 mm rotors prior to being placed into a full-length (17 mm), normal wall thickness (1 mm) Bruker 4 mm rotor. The inserts provide the advantage of restricting the sample length to the region of the coil in the NMR probe that has optimal RF homogeneity.

The peptide ((GPO)_5_(G*P*O)(GPO)_5_)_3_ and the ECM obtained from cell culture were lyophilised prior to packing into the Kel-F insert. The dry mass of the samples used for solid-state NMR experiments were 20.0 mg of pure peptide and 14.2 mg of ECM.

The mouse calvaria bone was not subject to any drying or dehydration procedures, and was simply broken into clips without cryomilling prior to packing into the Kel-F insert. We expect that this will lead to the bone sample being slightly more hydrated than the peptide or the cell culture ECM samples, though no discernible amounts of water was observed visually after the experiment, nor was a significant reduction in sample mass observed. 12.6 mg of mouse bone was used for solid-state NMR experiments.

### Solid-state NMR Spectroscopy

All solid-state NMR spectra were recorded on a Bruker Avance I NMR spectrometer with a 9.4 T superconducting magnet, operating at 400 MHz ¹H, 100 MHz ¹³C and 40 MHz ^15^N frequencies. For all experiments, magic angle spinning rate was set at 10 kHz, and the sample temperature is 297 K, unless otherwise specified.

^13^C cross-polarisation (^13^C CP) experiments: the standard cross polarisation sequence in the Bruker pulse programme library was used: ¹H 90° pulse length 2.5 μs, contact time 2.5 ms, with a ramped pulse on ¹H. During acquisition, SPINAL-64^35^ decoupling at 100 kHz was applied on ¹H.

2D ^13^C-^13^C double quantum (DQ)- single quantum (SQ) correlation NMR experiment: initial cross polarisation parameters were the same as in ^13^C CP experiments. At 10 kHz MAS, 70 kHz POST-C7 pulse sequence^36^ was applied on ^13^C to excite double quantum coherence in 0.4 ms. Magnetisation was returned to zero quantum by another 0.4 ms of POST-C7 sequence. During DQ evolution and reconversion, 100 kHz Lee-Goldburg decoupling was applied on ¹H. During acquisition 100 kHz SPINAL-64 decoupling was applied on ¹H. The pulse sequence used was an adapted version of the Avance I large sweep width POST-C7 experiment in the Bruker library. For *in vitro* ECM 128–256 scans and for the heavy mouse bone samples 432 scans per t_1_-slice were recorded.

2D ^13^C-^13^C proton-driven spin diffusion (PDSD) correlation NMR experiment: initial cross polarisation parameters were also the same as in ^13^C CP experiments. At 10 kHz MAS, the magnetisation was allowed to evolve at single-quantum coherence during the incremental delay, and returned to zero quantum coherence by a ^13^C 90° pulse with a length of 3.8 μs. ¹H decoupling was switched off during the mixing period to allow transfer of ^13^C magnetisation via dipolar coupling and spin diffusion^37^, with a ^13^C 90° readout pulse at the end of the mixing period. During both the incremental delay and acquisition periods, SPINAL-64 decoupling was applied at 100 kHz. The pulse sequence used was an adapted version of the Avance I CP spin diffusion experiment in the Bruker library. Mixing periods of between 5 and 200 ms were recorded. For *in vitro* ECM 64–400 scans (depending on the amount of sample) and for the heavy mouse bone samples 256 scans per t_1_-delay were recorded.

### Analysis of energy landscapes

The calculations employed a standard atomistic force field, namely Amber9 with implicit solvent (igb = 2), and the FF99SB parameter set^38^. In the computational potential energy landscape approach we employ geometry optimisation techniques to calculate local minima and the transition states and pathways that connect them, to construct a kinetic transition network^26^. The low energy region of the landscape was first sampled using basin-hopping global optimisation^39,40^. These minima were then connected using double-ended transition state searches, which identify new minima and pathways, progressively augmenting the database. Observable properties were extracted using standard methods of statistical mechanics and unimolecular rate theory, and the underlying database was refined using additional connection attempts until the quantities of interest appeared to have converged. Details of these procedures can be found in recent reviews^41,42^

The simulations conducted here, which seek to explain the results of the ssNMR data relating to flexibility of P_X_ prolines adjacent to hydroxyproline residues, required parameters for the Amber9 force field obtained from experimentally derived properties of hydroxyproline with post-translational modifications^43^. The initial starting points for (POG)_12_ were derived from PDB 1V7H^44,45^; those for (PAG)_12_ were generated from (POG)_12_ by replacing the hydroxyprolines with alanine.

For each of the two peptide sequences, (POG)_12_ and (PAG)_12_, 10000 geometry perturbations were applied to the equivalent PYG group (the 6th group of the trailing chain) in the central region of each collagen triple helix. The perturbed structures were then relaxed to local minima. The 10000 resulting minima were filtered to identify the unique structures, which correspond to the space of accessible conformations for the backbone in the given PGY triplets. The unique minima were then connected in further transition state searches, which helps to ensure that all the relevant structures in the accessible configuration space were located.

We ensured uniform exploration of the backbone dihedral space by randomly rotating rigid groups of atoms between carefully chosen atom pairs. For the (PAG)_12_ system the pairs were: the Cβ to Cδ axis of the proline tip, the Cα-Cα axis of the peptide bond between the proline and alanine, the N-C axis of the alanine residue, and the Cα to Cα axis of the peptide bond that trailed the alanine residue. This combination of rotation groups ensures that psi and phi dihedrals of the proline, the psi and phi dihedrals of the alanine, the phi angle of the trailing glycine, and the psi angle of the preceding glycine residue are sampled. In addition, the full range of endo and exo conformations of the P_X_ ring are encountered. For the (POG)_12_ system, the corresponding groups were chosen, except that the C-N rotation of the alanine was replaced by a Cβ-Cδ rotation of the hydroxyproline tip, again inducing exploration of the endo/exo states of the O_Y_ residue and corresponding backbone dihedrals.

The uniform sampling of the full set of backbone dihedral angles can generate local minima that include peptide bond rotations and stereochemical rotations, both of which do not correspond to a biologically-feasible collagen triple helix structure. For our analysis, we are only interested in dihedral angle distributions that can be found in a collagen triple helix^46,47^. Therefore, only those structures where all the perturbed residues relax into the range of phi = −50° to −90° and psi = 130° to 170° were accepted.

### Sequence alignment and analysis

For our analysis of the conservation of the GPO positions, we built multiple sequence alignments across diverse species for collagen α1(I) and α2(I) sequences. For each alpha chain, a set of orthologous sequences was acquired using the National Centre for Biotechnology Information (NCBI) Protein Database resources (https://www.ncbi.nlm.nih.gov/protein)^48^. The NCBI Reference Sequence Database (RefSeq)^49^ was used as the main resource for protein sequence data. A small proportion of the data was obtained from GenBank and the EMBL databases^50,51^. The initial sets of sequences identified from these databases were filtered to remove duplicates (including isoforms), leaving a single representative sequence for each species. Each set of sequences was aligned using the software program MUSCLE (http://www.drive5.com/muscle/)^52,53^. The final sequence sets used can be found in Supplementary Table S2–S4.

Conservation of each GPO triplet was determined by calculating the probability of the occurrence of the GPO triplet across all aligned sequences. The amino acids with the highest frequency at each alignment position was used to generate a consensus sequence.

To analyze the effect of missense mutations on GPO sites, we used the consensus sequences generated in the same procedure as described above. We mapped mutation sites reported for human variants into the consensus sequences and assessed how they align with conserved GPO positions on the collagen type I primary sequence. The National Centre for Biotechnology Information (NCBI) ClinVar database (https://www.ncbi.nlm.nih.gov/clinvar/) was used as a mutation data resource^34^. The search for mutation sites across procollagen alpha chains was performed using the relevant gene names and the following criteria: molecular consequence: missense mutation; variation type: single nucleotide; review status: at least one star. The GPO triplet is counted as being present at a mutation site if one position of any of its residues (G, P or O) overlaps with a reported mutation site. The analysis of correlation between mutation sites and conserved GPO locations was carried out for the major fibrillar alpha chains (type I, II and III) and for collagen type V alpha chains.

## Electronic supplementary material

Supplementary Information

Consensus sequence for collagen type I (all species)

## Data Availability

The datasets generated during and/or analysed during the current study will be available on the University of Cambridge Open Data portal upon publication (https://www.research-operations.admin.cam.ac.uk/policies/open-data); a link to the data will be provided for the published manuscript.
