# Supplementary material for: Proline provides site-specific flexibility for in vivo collagen
Source: Sci Rep. 2018 Sep 14;8:13809. doi: 10.1038/s41598-018-31937-x (PMC6138679; doi:10.1038/s41598-018-31937-x)
Supplement: Supplementary file 1 — Supplementary Information [file 41598_2018_31937_MOESM1_ESM.pdf]

Supplementary Information: Proline provides site-specific flexibility for in vivo collagen

Wing Ying Chow[1‡], Chris Forman[1], Dominique Bihan[2], Anna Puszkarska[1], Rakesh Rajan[1], David G. Reid[1], David A. Slatter[2], Lucy J. Colwell[1], David J. Wales[1], Richard W. Farndale[2], Melinda J. Duer[1*]

[1]Department of Chemistry, University of Cambridge, Lensfield Road, Cambridge CB2 1EW UK
[2]Department of Biochemistry, University of Cambridge, Downing Site, Cambridge CB2 1QW, UK

Corresponding author: Professor Melinda J Duer
Dept of Chemistry, University of Cambridge, Lensfield Road, Cambridge CB2 1EW UK
E-mail: mjd13@cam.ac.uk

[‡]Present address: Leibniz Forschungsinstitut für Molekulare Pharmakologie (FMP) im Forschungsverbund Berlin e.V., Campus Berlin-Buch, Robert-Rössle-Str 10, 13125 Berlin, Germany

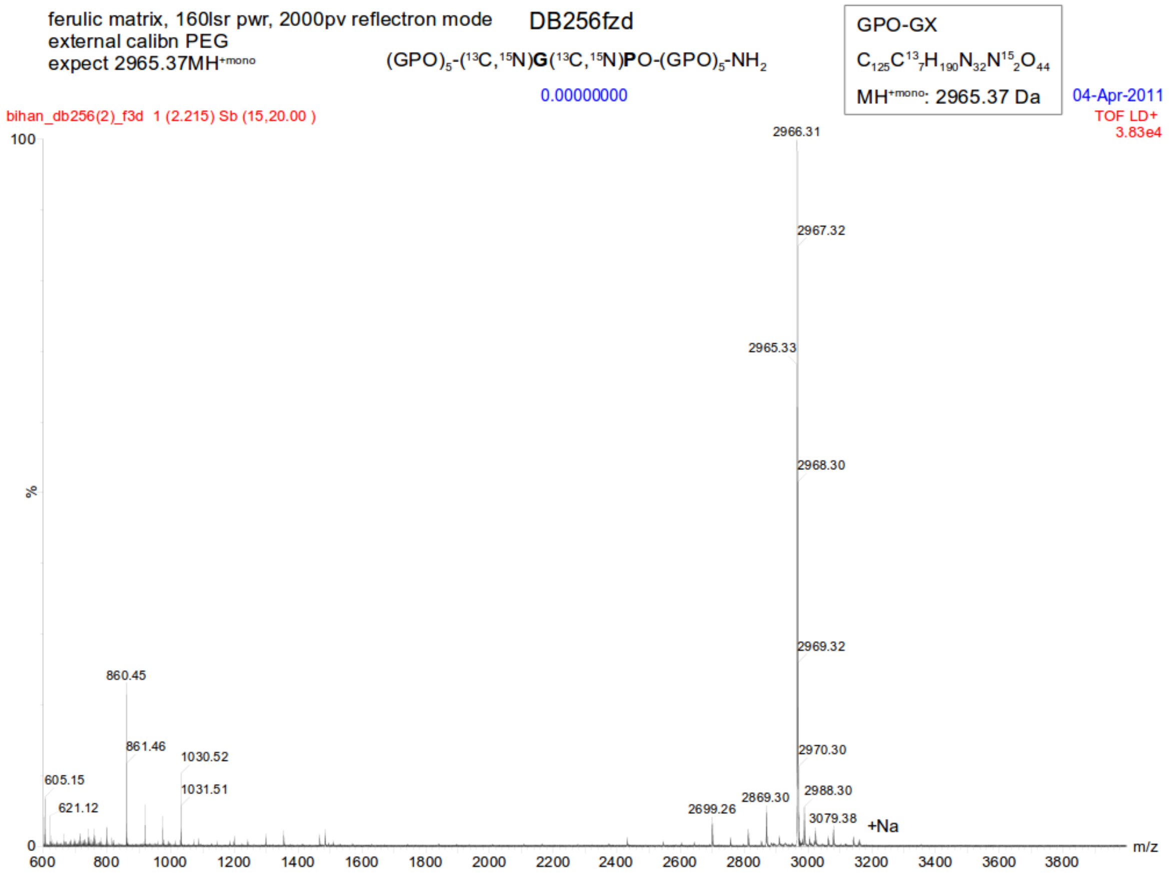

Supplementary Figure S1: Mass spectrum of the peptide $((GPO)_5(G^*P^*O)(GPO)_5)_3$ used in this study.

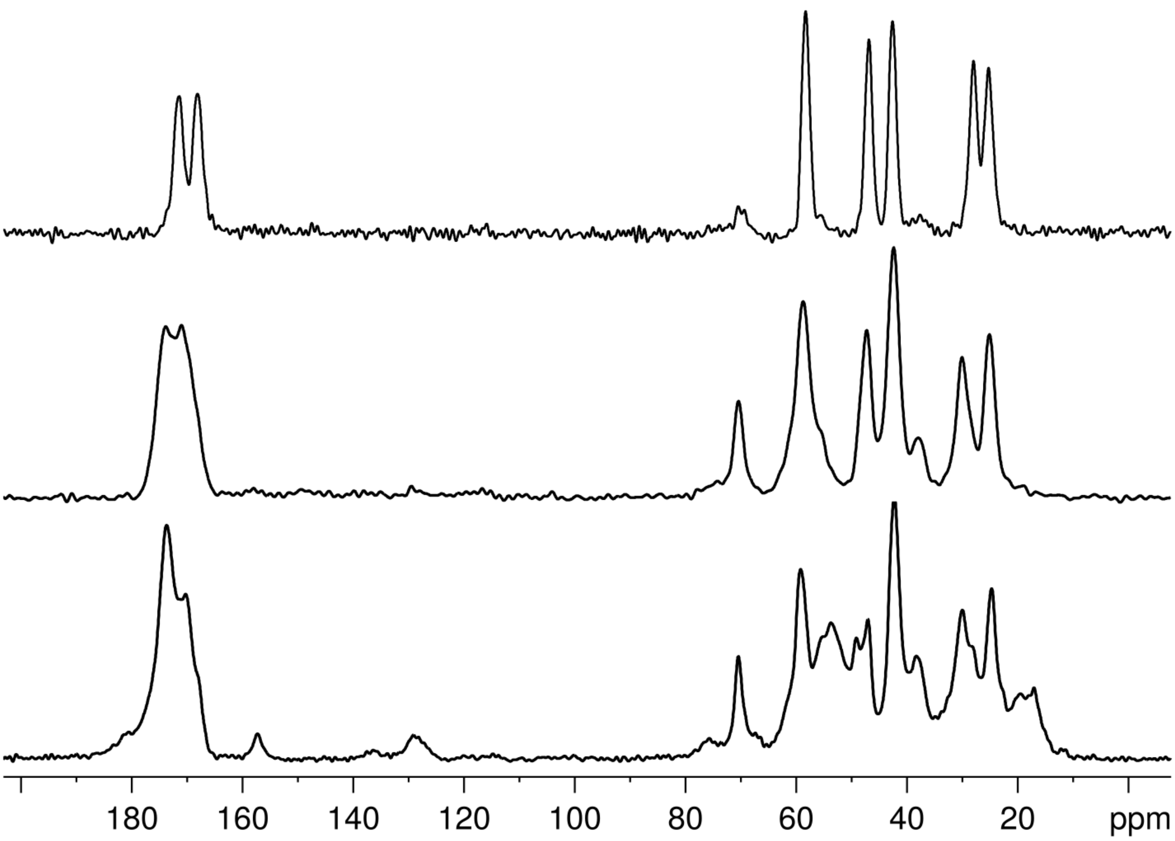

Supplementary Figure S2: ^{13}C CPMAS NMR spectrum of the collagen-like model peptide $((GPO)_5(G^*P^*O)(GPO)_5)_3$ (top), in vitro foetal sheep osteoblast extracellular matrix grown in medium containing U-^{13}C, ^{15}N-Gly, Pro (middle) and mouse bone containing ~20 % U-^{13}C, ^{15}N-labelled essential amino acids and glycine (bottom).

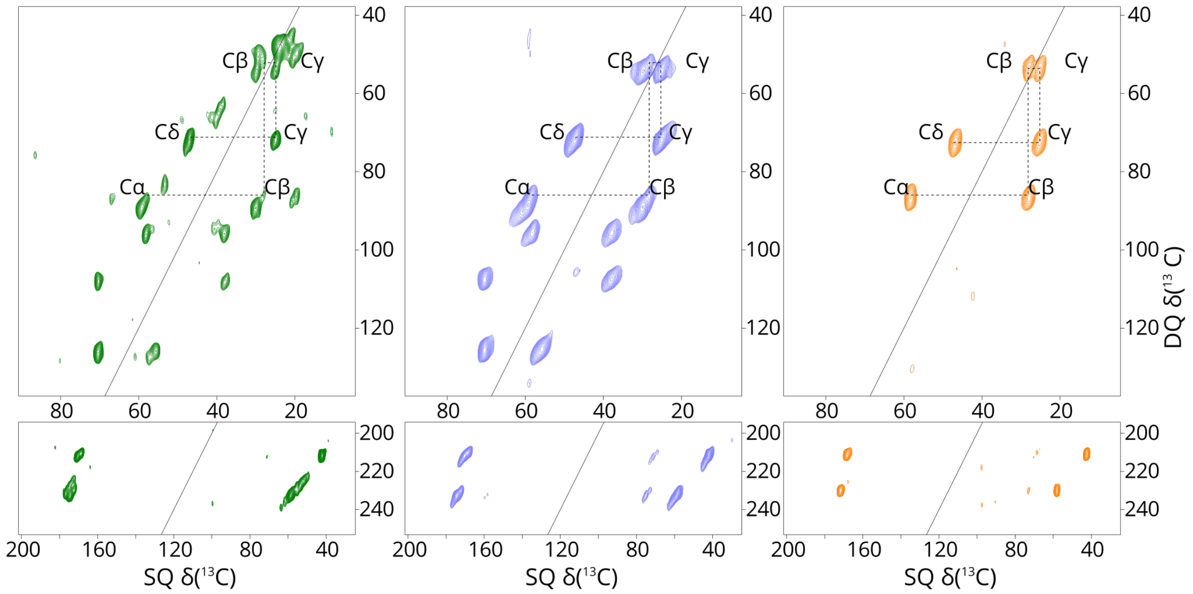

Supplementary Figure S3: 2D ^{13}C-^{13}C DQ–SQ correlation spectra of mouse calvarial bone (green), in vitro osteoblast extracellular matrix (blue) and model collagen peptide (orange), shown with the full chemical shift range.

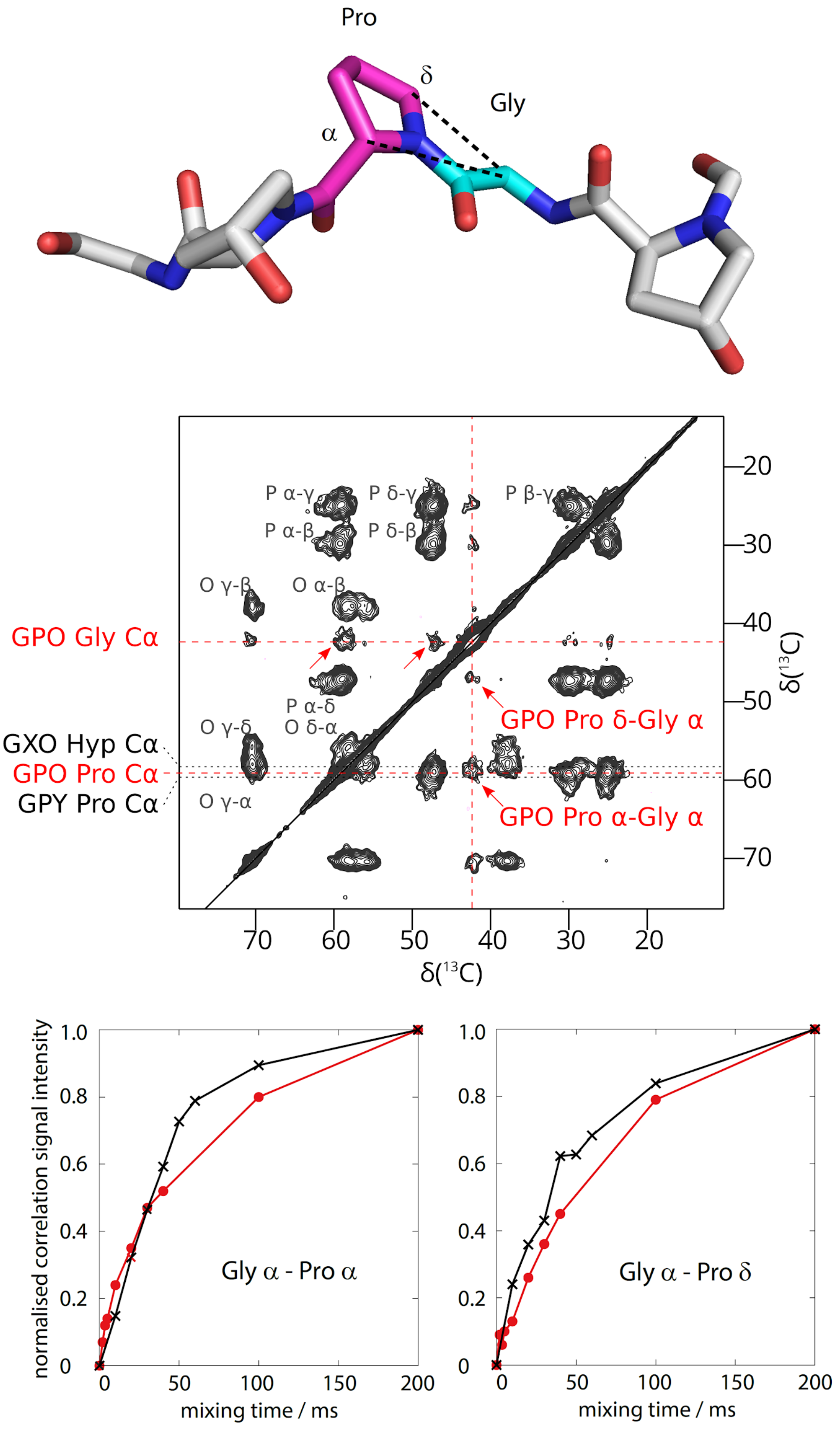

Supplementary Figure S4: Using PDSD to assign GPO P_X signals and confirming a similar molecular conformation. Top: part of the crystal structure of $(POG)_{10}$ (PDB 1V6Q) [1] illustrating the structure of a GPO triplet (OGPO is shown). Middle: 2D ^{13}C-^{13}C PDSD spectrum of U-^{13}C, ^{15}N-Pro, Hyp, Gly labelled foetal sheep osteoblast ECM with 70 ms mixing time at 10 kHz MAS. The proline and hydroxyproline intra-residue correlation signals are assigned, along with inter-residue correlation signals between GPO P_X Cα and Gly Cα (red arrows).

The C′, Cα and Cβ signals for proline in GPO triplets are shifted to lower frequency relative to proline carbons in general GPY triplets, due to the "proline effect" of hydroxyproline (O) in GPO triplets [1,2,3] on the preceding residue, in this case P_X, and this assigns the GPO P_X Cα, Cβ and C′. GPO Cδ is assigned by correlation with the GPO Cα. Bottom: Correlation signal intensity build-up from ^{13}C-^{13}C PDSD experiments with increasing mixing times for the GPO Gly Cα - Pro Cα and Gly Cα - Pro Cδ correlation signals. Red line is for the sheep osteoblast ECM sample, and black line is for the peptide sample $((GPO)_5(G^*P^*O)(GPO)_5)_3$.

The rate of cross peak intensity build-up in PDSD magnetization transfer curves depends on the ^{13}C-^{13}C dipolar coupling for the particular pair of correlated C spins and on ^{13}C-^{1}H dipolar couplings affecting either of the two ^{13}C nuclei; thus the rate of intensity build-up depends on the geometry of the ^{13}C-^{1}H spin system for the two nuclei concerned. Thus we can use inter-residue correlations between Gly and Pro(X) in GPO triplets as a probe to compare the geometries of the Gly-Pro(X) moiety of GPO triplets in bone collagen and the model collagen peptide.

In the final part of Supplementary Fig. S4, we show the PDSD intensity build-up curves for the in vitro collagen ECM sample (red) overlaid with those for the model peptide (black), for two interresidue distances between glycine and proline in GPO triplets, namely Gly Cα - Pro Cα and Gly Cα - Pro Cδ. The aim of this comparison is to show that the two buildup curves are qualitatively fairly similar, especially in the start of the buildup curve (0-50 μs), and not to provide quantitative estimations of the buildup rate. As pointed out in the main text, we expect that the in vitro ECM sample to exhibit a much wider range of conformations than that observed in a simple peptide. This comparison illustrates that the range of conformations observed in the in vitro ECM sample and the peptide are likely to be similar.

The in vitro collagen GPO P_X Cα signal is distinguished from the more intense GPY P_X signal by the proline effect as described above. The signal intensity for the Pro Cδ signals is taken as that at the corresponding chemical shift for GPO P_X measured in the DQ-SQ correlation spectra above; there will be some overlap of signal intensity from P_X rings in GPY, rather than GPO, triplets, but the intensity is dominated at these frequencies by the GPO P_X signals. These interresidue Gly Cα - Pro carbon correlations are only observed for GPO P_X, not for general GPY triplets, even though GPY triplets are more abundant than GPO.

The build up rates were normalised with respect to the 200 ms time signal intensity in both cases; it is assumed the correlation signal has reached maximum intensity by this time. If in fact this is not the case, normalization will underestimate the build-up rate. Thus the intensity build-up rates in shown in Supplementary Fig. S4 represent an upper limit. The build-up curves are also modulated by relaxation processes which may be different between the in vitro collagen and the model peptide. Accordingly, we focus on the initial slopes of the build-up curves where relaxation effects will be minimal for both samples. From visual inspection, we confirm that the initial slopes of the build-up curves for in vitro collagen and the model peptide are highly similar within the approximation made by the normalisation process. This result suggests similar Gly-Pro geometries for the model peptide and in vitro matrix collagen.

α1(I)							α2(I)						
positions of the conserved G-P-O triplets across the consensus sequence			triplet probability (p)	residue probability (p)			positions of the conserved G-P-O triplets across the consensus sequence			triplet probability (p)	residue probability (p)		
G	P	O		G	P	O	G	P	O		G	P	O
13	14	15	0.93	1	0.99	0.94	10	11	12	0.91	1	0.98	0.92
25	26	27	0.91	1	0.96	0.94							
46	47	48	0.98	1	0.98	1	46	47	48	0.89	1	0.91	0.96
67	68	69	0.83	1	0.95	0.86							
154	155	156	0.95	1	0.99	0.96							
163	164	165	0.93	1	0.98	0.95	163	164	165	0.96	1	0.98	0.98
196	197	198	0.76	1	0.78	0.96	205	206	207	0.85	1	0.99	0.86
280	281	282	0.97	1	0.99	0.975	280	281	282	0.8	1	0.89	0.9
388	389	390	0.88	1	0.99	0.88	442	443	444	0.81	1	0.93	0.83
391	392	393	0.82	1	0.96	0.82	460	461	462	0.79	1	0.8	0.98
424	425	426	0.76	1	0.9	0.76							
592	593	594	0.88	1	0.89	0.98	523	524	525	0.77	1	0.8	0.96
628	629	630	0.85	1	0.93	0.92							
637	638	639	0.98	1	0.99	0.99							
661	662	663	0.81	1	0.89	0.92							
691	692	693	0.99	1	1	0.99	691	692	693	0.85	1	0.98	0.87
706	707	708	0.99	1	0.99	0.99	706	707	708	0.83	1	0.85	0.93
715	716	717	0.76	1	0.79	0.97	715	716	717	0.93	1	0.99	0.93
718	719	720	0.88	1	0.95	0.93	718	719	720	0.79	1	0.92	0.79
748	749	750	0.97	1	0.98	0.98	748	749	750	0.88	1	0.96	0.9
817	818	819	0.98	1	0.99	0.98	766	767	768	0.85	1	0.86	0.97
823	824	825	0.99	1	0.99	1	811	812	813	0.79	1	0.9	0.83
829	830	831	0.88	1	0.88	1	844	845	846	0.92	1	0.93	0.99
865	866	867	0.8	1	0.8	0.95							
940	941	942	0.93	1	0.96	0.96							
943	944	945	0.81	1	0.93	0.88							
964	965	966	0.83	1	0.99	0.83							
988	989	990	0.99	1	0.99	1							
1000	1001	1002	0.94	1	0.98	0.96	1000	1001	1002	0.94	1	0.99	0.95
1003	1004	1005	0.97	1	0.98	0.99	1003	1004	1005	0.86	1	0.86	1
1006	1007	1008	0.96	1	0.98	0.98	1006	1007	1008	0.76	1	0.76	1
1009	1010	1011	0.94	1	0.94	1	1009	1010	1011	0.93	1	0.98	0.95

Supplementary Table S1: The GPO conservation analysis data. The table presents the exact positions across the α1(I) and α2(I) consensus sequences, where GPO triplets were found to be highly conserved. The calculated probability of the occurrence of the GPO triplets at particular sequence location as well as the estimated probabilities of corresponding residues are given for each collagen type I alpha chain. The background colour of the rows corresponds to the particular D-subdomain (D1 – blue, D2 – yellow, D3 – green, D4 – orange, D5 – violet). The highly conserved GPO positions, common for both alpha chains, have been marked in red (helix compression/extension sites, in text).

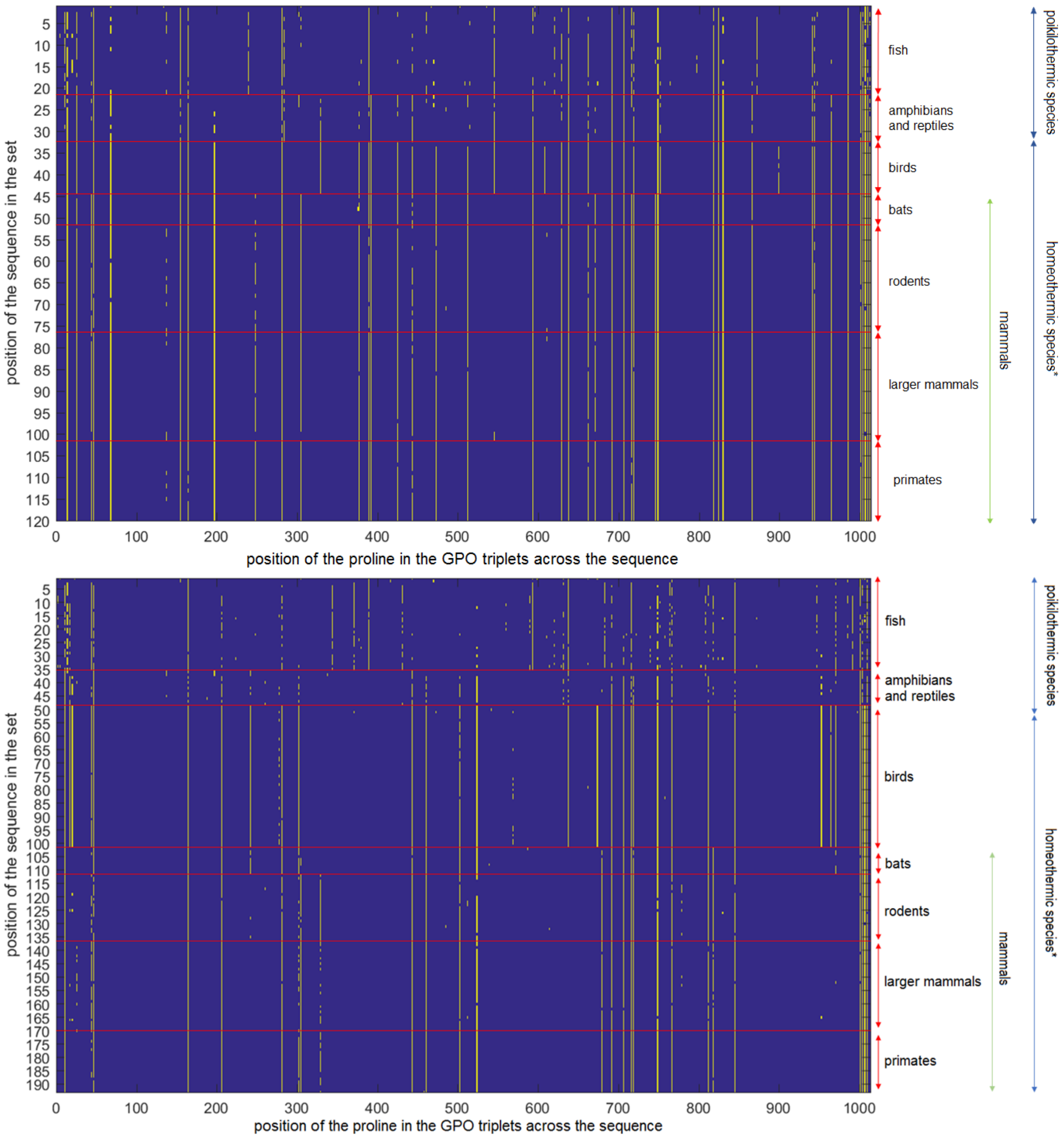

Supplementary Figure S5: Distribution of the GPO triplets across the triple helical region of collagen α1(I) (top) and collagen α2(I) (bottom) in various organisms. The diagram presents the correlation between the taxonomic affiliation of the species and the distribution of the GPO triplets across the helical region of the collagen type I sequences. For both chains, the distribution of the GPO triplets appear to be highly conserved across each homoeothermic group of species (birds, rodents, bats, larger mammals, primates), however, changes in the GPO locations are observed across them, particularly for α2(I) chain. Those GPO positions that are well-preserved across all species are given in Supplementary Table S1. Barring a few exceptions, variability in the GPO locations is predominantly observed among fish, amphibians and reptiles.

poikilothermic species	invertabrates		Insects
	vertabrates		Fish
			Amphibians
			Reptiles
			Birds
homoeothermic species	mammals		Bats
			Rodents
			Larger mammals
			Primates
			Human

Supplementary Table S2: Legend of colour code used in Supplementary Table S3 and Supplementary Table S4

The triple helix analysis presented in the main text contains 120 collagen α1(I) sequences and 193 collagen α2(I) chain sequences. The sequences in the alignments are ordered according to the taxonomy of the species, taking into account the phylum and the class, note this ordering has no effect on the conservation statistics reported in the main text. The ordering starts from the simplest multicellular organisms (*Hydra vulgaris*) and concludes with the higher vertebrates. The last sequence in each alignment is from human (*Homo sapiens*).

Position	Organism	Gene	Accession	Data base	Position	Organism	Gene	Accession	Data base
1	Hydra vulgaris	COL1A1	CDG71541	EMBL	61	Sorex araneus	COL1a1	XP_004608727	RefSeq
2	Coryphaenoides yaquinae	COL1A1	BAV03979	GeneBank	62	Condylura cristata	COL1a1	XP_004684395	RefSeq
3	Lepisosteus oculatus	COL1A1	XP_006638297	RefSeq	63	Marmota marmota marmota	COL1a1	XP_015350975	RefSeq
4	Hypophthalmichthys molitrix	COL1A1	AIL02135	EMBL	64	Ochotona princeps	COL1a1	XP_004591119	RefSeq
5	Anguilla japonica	COL1A1	BAP63924	GeneBank	65	Nannospalax galili	COL1A1	XP_008836559	RefSeq
6	Carassius auratus	COL1A1	BAG72200	GeneBank	66	Heterocephalus glaber	COL1A1	XP_004859739	RefSeq
7	Pygocentrus nattereri	COL1A1	XP_017540674	RefSeq	67	Chinchilla lanigera	COL1A1	XP_005394233	RefSeq
8	Fundulus heteroclitus	COL1A1	XP_012716616	RefSeq	68	Oryctolagus cuniculus	COL1A1	XP_017204320	RefSeq
9	Stegastes partitus	COL1A1	XP_008293593	RefSeq	69	Dipodomys ordii	COL1A1	XP_012893546	RefSeq
10	Paralichthys olivaceus	COL1A1	BAD77968	GeneBank	70	Octodon degus	COL1A1	XP_004633690	RefSeq
11	Oreochromis niloticus	COL1A1	NP_001266373	RefSeq	71	Fukomys damarensis	COL1A1	XP_010618446	RefSeq
12	Maylandia zebra	COL1A1	XP_004572575	RefSeq	72	Erinaceus europaeus	COL1A1	XP_007525671	RefSeq
13	Haplochromis burtoni	COL1A1	XP_005946024	RefSeq	73	Microcebus murinus	COL1A1	XP_012645195	RefSeq
14	Latimeria chalumnae	COL1A1	XP_005992246	RefSeq	74	Jaculus jaculus	COL1A1	XP_004655625	RefSeq
15	Poecilia mexicana	COL1A1	XP_014827396	RefSeq	75	Tupaia chinensis	COL1A1	XP_014438726	RefSeq
16	Poecilia formosa	COL1A1	XP_007565541	RefSeq	76	Cavia porcellus	COL1A1	XP_013004992	RefSeq
17	Pundamilia nyererei	COL1A1	XP_005750908	RefSeq	77	Sus scrofa domesticus	COL1A1	BAX02568	GeneBank
18	Acipenser schrenckii	COL1A1	BAR72356	GeneBank	78	Galeopterus variegatus	COL1A1	XP_008593281	RefSeq
19	Rhincodon typus	COL1A1	XP_020388689	RefSeq	79	Orycteropus afer afer	COL1A1	XP_007940107	RefSeq
20	Oncorhynchus mykiss	COL1A1	NP_001117649	RefSeq	80	Panthera pardus	COL1A1	XP_019271348	RefSeq
21	Danio rerio	COL1A1	NP_954684	RefSeq	81	Felis catus	COL1A1	XP_003996748	RefSeq
22	Xenopus tropicalis	COL1A1	NP_001011005	RefSeq	82	Odobenus rosmarus divergens	COL1A1	XP_004395207	RefSeq
23	Nanorana parkeri	COL1A1	XP_018408224	RefSeq	83	Canis lupus familiaris	COL1A1	NP_001003090	RefSeq
24	Xenopus laevis	COL1A1	NP_001080821	RefSeq	84	Equus asinus	COL1A1	NP_001310708	RefSeq
25	Gekko japonicus	COL1A1	XP_015269157	RefSeq	85	Orcinus orca	COL1A1	XP_004282670	RefSeq
26	Alligator sinensis	COL1A1	XP_006023387	RefSeq	86	Manis javanica	COL1A1	XP_017497917	RefSeq
27	Chrysemys picta bellii	COL1A1	XP_005301998	RefSeq	87	Bos taurus	COL1A1	NP_001029211	RefSeq
28	Protobothrops mucrosquamatus	COL1A1	XP_015665895	RefSeq	88	Bos indicus	COL1A1	XP_019836718	RefSeq
29	Pogona vitticeps	COL1A1	XP_020649626	RefSeq	89	Bison bison bison	COL1A1	XP_010841089	RefSeq
30	Anolis carolinensis	COL1A1	XP_016850114	RefSeq	90	Bos mutus	COL1A1	XP_005890387	RefSeq
31	Thamnophis sirtalis	COL1A1	XP_013916526	RefSeq	91	Loxodonta africana	COL1A1	XP_010592644	RefSeq
32	Python bivittatus	COL1A1	XP_007424908	RefSeq	92	Ovis aries musimon	COL1A1	XP_011983013	RefSeq
33	Alligator mississippiensis	COL1A1	XP_006277120	RefSeq	93	Capra hircus	COL1A1	XP_017920382	RefSeq
34	Pseudopodoces humilis	COL1a1	XP_014116476	RefSeq	94	Camelus dromedarius	COL1A1	XP_010996883	RefSeq
35	Coturnix japonica	COL1a1	XP_015741354	RefSeq	95	Camelus bactrianus	COL1A1	XP_010947400	RefSeq
36	Cuculus canorus	COL1a1	XP_009569448	RefSeq	96	Camelus ferus	COL1A1	XP_014406297	RefSeq
37	Aquila chrysaetos canadensis	COL1a1	XP_011592259	RefSeq	97	Carlito syrichta	COL1A1	XP_008063438	RefSeq
38	Lepidothrix coronata	COL1a1	XP_017694230	RefSeq	98	Vicugna pacos	COL1A1	XP_006218715	RefSeq
39	Haliaeetus leucocephalus	COL1a1	XP_010583768	RefSeq	99	Ailuropoda melanoleuca	COL1A1	XP_002923775	RefSeq
40	Nipponia nippon	COL1a1	XP_009462891	RefSeq	100	Monodelphis domestica	COL1A1	XP_001367735	RefSeq
41	Falco peregrinus	COL1a1	XP_005235901	RefSeq	101	Sarcophilus harrisii	COL1A1	XP_003768423	RefSeq
42	Falco cherrug	COL1a1	XP_005439463	RefSeq	102	Mandrillus leucophaeus	COL1A1	XP_011854657	RefSeq
43	Sturnus vulgaris	COL1a1	XP_014744888	RefSeq	103	Chlorocebus sabaeus	COL1A1	XP_008009716	RefSeq
44	Parus major	COL1a1	XP_015471795	RefSeq	104	Cercocebus atys	COL1A1	XP_011901567	RefSeq
45	Rhinolophus sinicus	COL1a1	XP_019586497	RefSeq	105	Gorilla gorilla gorilla	COL1A1	XP_004041373	RefSeq
46	Eptesicus fuscus	COL1a1	XP_008151928	RefSeq	106	Colobus angolensis palliatus	COL1A1	XP_011815340	RefSeq
47	Miniopterus natalensis	COL1a1	XP_016071097	RefSeq	107	Otolemur garnettii	COL1A1	XP_003786538	RefSeq
48	Myotis brandtii	COL1a1	XP_005868232	RefSeq	108	Propithecus coquereli	COL1A1	XP_012516812	RefSeq
49	Hipposideros armiger	COL1a1	XP_019493986	RefSeq	109	Aotus nancymaae	COL1A1	XP_012318928	RefSeq
50	Myotis lucifugus	COL1a1	XP_006105322	RefSeq	110	Rhinopithecus roxellana	COL1A1	XP_010384431	RefSeq
51	Pteropus alecto	COL1a1	XP_006924855	RefSeq	111	Rhinopithecus bieti	COL1A1	XP_017709381	RefSeq
52	Echinops telfairi	COL1a1	XP_004707116	RefSeq	112	Cebus capucinus imitator	COL1A1	XP_017395605	RefSeq
53	Rattus norvegicus	COL1a1	NP_445756	RefSeq	113	Macaca nemestrina	COL1A1	XP_011723843	RefSeq
54	Mus musculus	COL1a1	NP_031768	RefSeq	114	Saimiri boliviensis boliviensis	COL1A1	XP_003931313	RefSeq
55	Microtus ochrogaster	COL1a1	XP_005350656	RefSeq	115	Callithrix jacchus	COL1A1	JAB40423	GeneBank
56	Mustela putorius furo	COL1a1	XP_004764878	RefSeq	116	Macaca mulatta	COL1A1	XP_014974803	RefSeq
57	Castor canadensis	COL1a1	XP_020026973	RefSeq	117	Macaca fascicularis	COL1A1	XP_015293729	RefSeq
58	Mesocricetus auratus	COL1a1	XP_005075907	RefSeq	118	Pan paniscus	COL1A1	XP_003817507	RefSeq
59	Cricetulus griseus	COL1a1	XP_003504008	RefSeq	119	Pan troglodytes	COL1A1	XP_001169409	RefSeq
60	Peromyscus maniculatus bairdii	COL1a1	XP_006972005	RefSeq	120	Homo sapiens	COL1A1	NP_000079	RefSeq

Supplementary Table S3: Dataset of collagen α1(I) sequences used to determine collagen type I α1 consensus sequence and for the conservation analysis in Fig. 6. Positions of the sequences in the set have been indicated. Species have been ordered in the alignment according to their biological classification and the colour of the field corresponds to the specific group.

Position	Organism	Gene	Accesion	Data base	Position	Organism	Gene	Accesion	Data base
1	Rhincodon typus	COL1A2	XP_020371053	RefSeq	98	Serinus canaria	COL1A2	XP_009084209	RefSeq
2	Callorhinchus milii	COL1A2	XP_007907447	RefSeq	99	Cuculus canorus	COL1A1	XP_009557161	RefSeq
3	Salmo salar	COL1A2	XP_014033985	RefSeq	100	Columba livia	COL1A0	XP_005504983	RefSeq
4	Oreochromis niloticus	COL1A2	NP_001269826	RefSeq	101	Ficedula albicollis	COL1A1	XP_005041149	RefSeq
5	Maylandia zebra	COL1A2	XP_004547977	RefSeq	102	Rhinolophus sinicus	COL1A2	XP_019585276	RefSeq
6	Pundamilia nyererei	COL1A2	XP_005728787	RefSeq	103	Eptesicus fuscus	COL1A2	XP_008147219	RefSeq
7	Haplochromis burtoni	COL1A2	XP_005928634	RefSeq	104	Miniopterus natalensis	COL1A2	XP_016074330	RefSeq
8	Poecilia formosa	COL1A2	XP_007573076	RefSeq	105	Hipposideros armiger	COL1A2	XP_019480273	RefSeq
9	Poecilia mexicana	COL1A2	XP_014852900	RefSeq	106	Pteropus alecto	COL1A2	XP_015444911	RefSeq
10	Poecilia latipinna	COL1A2	XP_014895871	RefSeq	107	Pteropus vampyrus	COL1A2	XP_011361841	RefSeq
11	Stegastes partitus	COL1A2	XP_008287498	RefSeq	108	Rousettus aegyptiacus	COL1A2	XP_016016355	RefSeq
12	Cynoglossus semilaevis	COL1A2	XP_008329548	RefSeq	109	Myotis davidii	COL1A2	XP_015413820	RefSeq
13	Notothenia coriiceps	COL1A2	XP_010772950	RefSeq	110	Myotis brandtii	COL1A2	XP_005876138	RefSeq
14	Oryzias latipes	COL1A2	XP_011479246	RefSeq	111	Myotis lucifugus	COL1A2	XP_006085025	RefSeq
15	Takifugu rubripes	COL1A2	XP_011607785	RefSeq	112	Dipodomys ordii	COL1A2	XP_012881513	RefSeq
16	Fundulus heteroclitus	COL1A2	XP_012707153	RefSeq	113	Octodon degus	COL1A2	XP_004632680	RefSeq
17	Austrofundulus limnaeus	COL1A2	XP_013866709	RefSeq	114	Rattus norvegicus	COL1A2	NP_445808	RefSeq
18	Lates calcarifer	COL1A2	XP_018522130	RefSeq	115	Mus musculus	COL1A2	NP_031769	RefSeq
19	Xiphophorus maculatus	COL1A2	XP_014325625	RefSeq	116	Peromyscus maniculatus bairdii	COL1A2	XP_006991260	RefSeq
20	Larimichthys crocea	COL1A2	XP_019118204	RefSeq	117	Microtus ochrogaster	COL1A2	XP_005363739	RefSeq
21	Kryptolebias marmoratus	COL1A2	XP_017279795	RefSeq	118	Mesocricetus auratus	COL1A2	XP_005082957	RefSeq
22	Latimeria chalumnae	COL1A2	XP_006011686	RefSeq	119	Erinaceus europaeus	COL1A2	XP_007522561	RefSeq
23	Danio rerio	COL1A2	NP_892013	RefSeq	120	Nannospalax galili	COL1A2	XP_008820781	RefSeq
24	Oncorhynchus mykiss	COL1A2	NP_001117679	RefSeq	121	Sorex araneus	COL1A2	XP_004602244	RefSeq
25	Paralichthys olivaceus	COL1A2	XP_019946222	RefSeq	122	Ochotona princeps	COL1A2	XP_004582363	RefSeq
26	Esox lucius	COL1A2	XP_010889809	RefSeq	123	Oryctolagus cuniculus	COL1A2	NP_001182597	RefSeq
27	Ictalurus punctatus	COL1A2	XP_017309977	RefSeq	124	Condylura cristata	COL1A2	XP_004676750	RefSeq
28	Clupea harengus	COL1A2	XP_012685017	RefSeq	125	Echinops telfairi	COL1A2	XP_004702721	RefSeq
29	Hippocampus comes	COL1A2	XP_019745275	RefSeq	126	Cavia porcellus	COL1A2	XP_005006094	RefSeq
30	Scleropages formosus	COL1A2	XP_018587334	RefSeq	127	Mustela putorius furo	COL1A2	XP_004767919	RefSeq
31	Lepisosteus oculatus	COL1A2	XP_015213472	RefSeq	128	Heterocephalus glaber	COL1A2	XP_004862750	RefSeq
32	Sinocyclocheilus grahami	COL1A2	XP_016138752	RefSeq	129	Castor canadensis	COL1A2	XP_020019043	RefSeq
33	Pygocentrus nattereri	COL1A2	XP_017550401	RefSeq	130	Jaculus jaculus	COL1A2	XP_004656276	RefSeq
34	Cyprinodon variegatus	COL1A2	XP_015231985	RefSeq	131	Fukomys damarensis	COL1A2	XP_010601746	RefSeq
35	Nothobranchius furzeri	COL1A2	XP_015811330	RefSeq	132	Ictidomys tridecemlineatus	COL1A2	XP_005331878	RefSeq
36	Xenopus tropicalis	COL1A2	NP_001072718	RefSeq	133	Marmota marmota marmota	COL1A2	XP_015343716	RefSeq
37	Xenopus laevis	COL1A2	NP_001080727	RefSeq	134	Cricetulus griseus	COL1A2	XP_003497018	RefSeq
38	Alligator sinensis	COL1A2	XP_006025957	RefSeq	135	Chinchilla lanigera	COL1A2	XP_005388098	RefSeq
39	Anolis carolinensis	COL1A2	XP_008110742	RefSeq	136	Tupaia chinensis	COL1A2	XP_006157541	RefSeq
40	Python bivittatus	COL1A2	XP_007425176	RefSeq	137	Panthera pardus	COL1A2	XP_019298880	RefSeq
41	Alligator mississippiensis	COL1A2	XP_006258514	RefSeq	138	Canis lupus familiaris	COL1A2	NP_001003187	RefSeq
42	Gavialis gangeticus	COL1A2	XP_019363980	RefSeq	139	Orcinus orca	COL1A2	XP_004265635	RefSeq
43	Gekko japonicus	COL1A2	XP_015275703	RefSeq	140	Odobenus rosmarus divergens	COL1A2	XP_004394135	RefSeq
44	Crocodylus porosus	COL1A2	XP_019388912	RefSeq	141	Dasypus novemcinctus	COL1A2	XP_004470764	RefSeq
45	Pelodiscus sinensis	COL1A2	XP_006114551	RefSeq	142	Bos taurus	COL1A2	NP_776945	RefSeq
46	Thamnophis sirtalis	COL1A2	XP_013912965	RefSeq	143	Felis catus	COL1A2	XP_003982813	RefSeq
47	Chrysemys picta bellii	COL1A2	XP_005308617	RefSeq	144	Ovis aries	COL1A2	XP_004007775	RefSeq
48	Protobothrops mucrosquamatus	COL1A2	XP_015667060	RefSeq	145	Ailuropoda melanoleuca	COL1A2	XP_002918275	RefSeq
49	Egretta garzetta	COL1A2	XP_009640166	RefSeq	146	Equus caballus	COL1A2	XP_001492989	RefSeq
50	Calidris pugnax	COL1A2	XP_014795757	RefSeq	147	Sus scrofa	COL1A2	NP_001230584	RefSeq
51	Calypte anna	COL1A2	XP_008492371	RefSeq	148	Equus asinus	COL1A2	NP_001310709	RefSeq
52	Balearica regulorum gibbericeps	COL1A2	XP_010298449	RefSeq	149	Pantholops hodgsonii	COL1A2	XP_005985745	RefSeq
53	Sturnus vulgaris	COL1A2	XP_014731120	RefSeq	150	Capra hircus	COL1A2	XP_005678993	RefSeq
54	Coturnix japonica	COL1A2	XP_015709029	RefSeq	151	Bubalus bubalis	COL1A2	XP_006054012	RefSeq
55	Pseudopodoces humilis	COL1A2	XP_005518863	RefSeq	152	Carlito syrichta	COL1A2	XP_008067045	RefSeq
56	Cariama cristata	COL1A2	XP_009707296	RefSeq	153	Loxodonta africana	COL1A2	XP_010585560	RefSeq
57	Corvus brachyrhynchos	COL1A2	XP_008628130	RefSeq	154	Bison bison bison	COL1A2	XP_010838069	RefSeq
58	Geospiza fortis	COL1A2	XP_005418631	RefSeq	155	Bos indicus	COL1A2	XP_019815249	RefSeq
59	Mesitornis unicolor	COL1A2	XP_010181131	RefSeq	156	Panthera tigris altaica	COL1A2	XP_007076757	RefSeq
60	Tinamus guttatus	COL1A2	XP_010210602	RefSeq	157	Camelus bactrianus	COL1A2	XP_010970903	RefSeq
61	Phaethon lepturus	COL1A2	XP_010280840	RefSeq	158	Camelus dromedarius	COL1A2	XP_010977966	RefSeq
62	Aquila chrysaetos canadensis	COL1A2	XP_011583435	RefSeq	159	Manis javanica	COL1A2	XP_017517878	RefSeq
63	Parus major	COL1A2	XP_015475122	RefSeq	160	Tursiops truncatus	COL1A2	XP_019783093	RefSeq
64	Corvus cornix cornix	COL1A2	XP_010397702	RefSeq	161	Acinonyx jubatus	COL1A2	XP_014922022	RefSeq
65	Antrostomus carolinensis	COL1A2	XP_010176198	RefSeq	162	Equus asinus	COL1A2	XP_014708845	RefSeq
66	Haliaeetus albicilla	COL1A2	XP_009929196	RefSeq	163	Ursus maritimus	COL1A2	XP_008684476	RefSeq
67	Haliaeetus leucocephalus	COL1A2	XP_010568320	RefSeq	164	Equus przewalskii	COL1A2	XP_008522480	RefSeq
68	Picoides pubescens	COL1A2	XP_009905768	RefSeq	165	Galeopterus variegatus	COL1A2	XP_008569871	RefSeq
69	Gallus gallus	COL1A2	NP_001073182	RefSeq	166	Orycteropus afer afer	COL1A2	XP_007937996	RefSeq
70	Taeniopygia guttata	COL1A2	XP_012426898	RefSeq	167	Leptonychotes weddellii	COL1A2	XP_006730730	RefSeq
71	Anser cygnoides domesticus	COL1A2	XP_013042484	RefSeq	168	Vicugna pacos	COL1A2	XP_006207690	RefSeq
72	Apteryx australis mantelli	COL1A2	XP_013796745	RefSeq	169	Camelus ferus	COL1A2	XP_006178597	RefSeq
73	Zonotrichia albicollis	COL1A2	XP_014125656	RefSeq	170	Bos mutus	COL1A2	XP_005909757	RefSeq
74	Leptosomus discolor	COL1A2	XP_009951637	RefSeq	171	Microcebus murinus	COL1A2	XP_012612577	RefSeq
75	Lepidothrix coronata	COL1A2	XP_017673134	RefSeq	172	Propithecus coquereli	COL1A2	XP_012503150	RefSeq
76	Opisthocomus hoazin	COL1A2	XP_009934436	RefSeq	173	Colobus angolensis palliatus	COL1A2	XP_011810470	RefSeq
77	Tauraco erythrolophus	COL1A2	XP_009983928	RefSeq	174	Saimiri boliviensis boliviensis	COL1A2	XP_003921263	RefSeq
78	Chaetura pelagica	COL1A2	XP_009993210	RefSeq	175	Rhinopithecus roxellana	COL1A2	XP_010387823	RefSeq
79	Tyto alba	COL1A2	XP_009963611	RefSeq	176	Nomascus leucogenys	COL1A2	XP_004089605	RefSeq
80	Nestor notabilis	COL1A2	XP_010015343	RefSeq	177	Callithrix jacchus	COL1A2	XP_003733643	RefSeq
81	Charadrius vociferus	COL1A2	XP_009893653	RefSeq	178	Mandrillus leucophaeus	COL1A2	XP_011830060	RefSeq
82	Chlamydotis macqueenii	COL1A2	XP_010125621	RefSeq	179	Cercocebus atys	COL1A2	XP_011929270	RefSeq
83	Pterocles gutturalis	COL1A2	XP_010075677	RefSeq	180	Papio anubis	COL1A2	XP_003896357	RefSeq
84	Eurypyga helias	COL1A2	XP_010158799	RefSeq	181	Pongo abelii	COL1A2	XP_002818294	RefSeq
85	Fulmarus glacialis	COL1A2	XP_009573423	RefSeq	182	Macaca fascicularis	COL1A2	XP_005550260	RefSeq
86	Gavia stellata	COL1A2	XP_009806503	RefSeq	183	Macaca nemestrina	COL1A2	XP_011729152	RefSeq
87	Anas platyrhynchos	COL1A2	XP_005010990	RefSeq	184	Macaca mulatta	COL1A2	NP_001253266	RefSeq
88	Falco cherrug	COL1A2	XP_005432180	RefSeq	185	Rhinopithecus bieti	COL1A2	XP_017710454	RefSeq
89	Falco peregrinus	COL1A2	XP_005228841	RefSeq	186	Cebus capucinus imitator	COL1A2	XP_017380605	RefSeq
90	Aptenodytes forsteri	COL1A2	XP_009284612	RefSeq	187	Aotus nancymaae	COL1A2	XP_012306220	RefSeq
91	Pygoscelis adeliae	COL1A2	XP_009328782	RefSeq	188	Otolemur garnettii	COL1A2	XP_003782725	RefSeq
92	Nipponia nippon	COL1A2	XP_009464876	RefSeq	189	Chlorocebus sabaeus	COL1A2	XP_007980329	RefSeq
93	Manacus vitellinus	COL1A2	XP_008919434	RefSeq	190	Gorilla gorilla gorilla	COL1A2	XP_004045817	RefSeq
94	Struthio camelus australis	COL1A2	XP_009672566	RefSeq	191	Pan paniscus	COL1A2	XP_003809763	RefSeq
95	Apaloderma vittatum	COL1A2	XP_009865826	RefSeq	192	Pan troglodytes	COL1A2	XP_001168894	RefSeq
96	Pelecanus crispus	COL1A2	XP_009490310	RefSeq	**193**	Homo sapiens	COL1A2	NP_000080	RefSeq
97	Merops nubicus	COL1A2	XP_008948217	RefSeq					

Supplementary Table S4: Dataset of collagen α2(I) sequences used to determine collagen type I α2 consensus sequence and for the conservation analysis in Fig. 6. Positions of the sequences in the set have been indicated. Species have been ordered in the alignment according to their biological classification and the colour of the field corresponds to the specific group.

References

1. Okuyama, K. *et al.* Crystal structures of collagen model peptides with Pro-Hyp-Gly repeating sequence at 1.26 A resolution: implications for proline ring puckering. *Biopolymers* **76,** 367–377 (2004).

3. Wang, Y. & Jardetzky, O. Investigation of the Neighboring Residue Effects on Protein Chemical Shifts. *J. Am. Chem. Soc.* **124,** 14075–14084 (2002).

3. Wishart, D. S., Bigam, C. G., Holm, A., Hodges, R. S. & Sykes, B. D. 1H, 13C and 15N random coil NMR chemical shifts of the common amino acids. I. Investigations of nearest-neighbor effects. *J. Biomol. NMR* **5,** 67–81 (1995).
