# Supplementary material for: Proline provides site-specific flexibility for in vivo collagen
Source: Sci Rep. 2018 Sep 14;8:13809. doi: 10.1038/s41598-018-31937-x (PMC6138679; doi:10.1038/s41598-018-31937-x)
Supplement: Supplementary file 2 — Consensus sequence for collagen type I (all species) [file 41598_2018_31937_MOESM2_ESM.pdf]

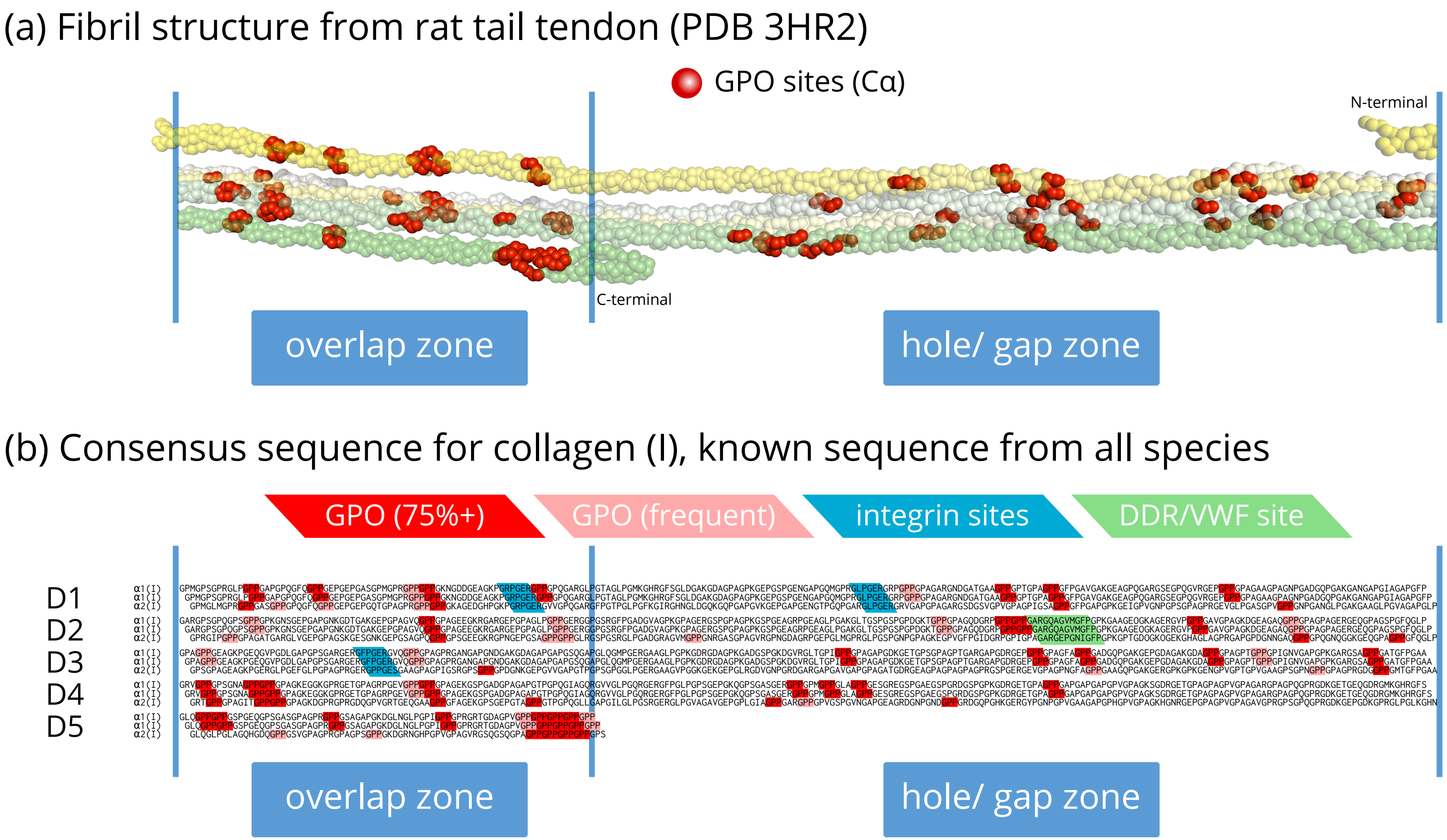

(a) Fibril structure from rat tail tendon (PDB 3HR2)
GPO sites (Cα)
N-terminal
C-terminal
overlap zone
hole/ gap zone
(b) Consensus sequence for collagen (I), known sequence from all species
GPO (75%+)
GPO (frequent)
integrin sites
DDR/VWF site
D1
D2
D3
D4
D5
α1(I)
α1(I)
α2(I)
overlap zone
hole/ gap zone
